# How Preferences and Reality on Where We Die Unfold: A Four‐Country Longitudinal Qualitative Study (EOLinPLACE)

**DOI:** 10.1111/hex.70732

**Published:** 2026-07-03

**Authors:** Sifra Hannah van de Beek, Krista Ann Eckels, Dorothy Adong Olet, Inês Dias da Silva, Mayra Delalibera, Joanna Veazey Brooks, Elizabeth Namukwaya, Jenny Theodora van der Steen, Yvette Milene van der Linden, Rui Garcia, Barbara Gomes, Dorothea Petra Touwen

**Affiliations:** ^1^ Faculty of Medicine University of Coimbra, Azinhaga de Santa Comba Coimbra Portugal; ^2^ Department of Medical Ethics and Health Law Leiden University Medical Center Leiden ZA the Netherlands; ^3^ Department of Occupational Therapy University of Kansas Medical Center Kansas City KS USA; ^4^ Institute of Hospice and Palliative Care in Africa, Hospice Africa Uganda Kampala Uganda; ^5^ Department of Population Health and Palliative Medicine University of Kansas School of Medicine Kansas City KS USA; ^6^ University of Kansas, Cancer Center Kansas City KS USA; ^7^ Department of Medicine, School of Medicine, College of Health Sciences Makerere University Kampala Uganda; ^8^ Palliative Care Education and Research Consortium Uganda; ^9^ Cicely Saunders Institute of Palliative Care, Policy and Rehabilitation, King's College London London UK; ^10^ Department of Public Health and Primary Care Leiden University Medical Center Leiden ZD the Netherlands; ^11^ Radboudumc Alzheimer Center and Department of Primary and Community Care Radboud university medical center Nijmegen GA the Netherlands; ^12^ Center of Expertise in Palliative Care Leiden University Medical Center Leiden ZA the Netherlands; ^13^ Netherlands Comprehensive Cancer Organisation (IKNL) Utrecht CV the Netherlands; ^14^ Palliative Care Service, Coimbra Health – Integrated Delivery System, Praceta Prof. Mota Pinto Coimbra Portugal

## Abstract

**Context:**

While there is a growing body of evidence on end‐of‐life (EOL) care preferences such as place of death, research remains limited in key areas. This includes gaps in understanding preferred and actual places of EOL care and death (dying places), potential shifts of preferences over time, and their (non‐)alignment with reality. We aimed to explore how preferred and actual dying places unfold for adults with life‐threatening illness and their family caregivers in different socio‐cultural settings.

**Methods:**

A qualitative longitudinal study in the Netherlands, Portugal, Uganda, and the United States (June 2023–August 2025) in adults (≥ 18 y) with cancer, dementia, neuromuscular or heart and cerebrovascular disease and their family caregivers. We conducted a semi‐structured interview at inclusion, followed by at least 2 interviews (between 3 weeks to 18 months after), including post‐death with the family caregiver. Fieldnotes of informal conversations and observations complemented the transcripts. Analysis was based on principles of applied qualitative ethnography, combining applied thematic analysis with thematic network analysis.

**Results:**

Fourteen patients participated, eight of whom were followed until death. Home was the most preferred dying place. We identified 3 themes: (1) *Beyond the preferred: choosing otherwise* highlighted how factors (the burden of receiving care, anticipated trauma of death at home, and urgent care needs) drove decisions around place; (2) *Family caregiver commitment and burden affecting realisation of patient preferences* illustrated the critical role of family caregivers; and (3) *Navigating care shapes dying places* showed how the healthcare system and skills to navigate it, influenced dying places.

**Discussion:**

Preferences and decisions were influenced by a complex interplay of personal, relational, and contextual considerations. The prominence of these considerations may vary by country, but their interaction and the way they shape preferred and actual dying places appear to be a shared phenomenon. Clinicians, policymakers, educators, and researchers must consider patients’ and family caregivers’ preferences along with influencing drivers that can support or limit choice.

**Patient or Public Contribution:**

Patient and public involvement and engagement (PPIE) was embedded in this international project from the start, through formal partnerships established with two international organisations representing patients and informal carers, namely the International Alliance of Patients’ Organisations (IAPO) and Eurocarers. Representatives of both organisations worked closely with the research team and are members of the project advisory board. They contributed to the development of the study design and materials, to the training of researchers helping ensure interviews with patients and family caregivers were conducted in a sensitive and appropriate manner, and to the interpretation of findings through various meetings. They will also help to disseminate the findings to engage patients, informal carers and the wider public.

## 1 Introduction

There is a growing body of evidence on end‐of‐life (EOL) preferences such as the places where people wish to die [1, 2, 3, 4, 5, 6, 7, 8]. Most people prefer dying at home for proximity to loved ones, maintaining autonomy, feelings of comfort, and seeking to avoid negative aspects of other locations than home [1, 7]. Hence, home is the prioritised place of death and has become a qualifier of a ‘good’ death compared to other places [6, 9, 10]. Statistics about place of death are being monitored by policy‐making bodies such as the Organisation for Economic Co‐operation and Development (OECD). The OECD raised the question of why care at the EOL is not matching people's preferences, since most people die in inpatient facilities (e.g., hospitals) [11, 12]. The answer appears complex. Studies show that place of death is determined by patient preference but also by multiple other factors [13, 14, 15]. There are clinical and healthcare‐related factors such as symptom control, functional status, formal support including home care and its intensity, hospice or palliative care enrolment, accessibility and quality of care, and rehospitalization [1, 3, 4, 9]. Also, personal and relational factors such as anxiety, values such as dignity and autonomy, the desire to be with loved ones, worries about being a burden, quality of life, cultural aspects, (not) having a support system, family caregiving ability and willingness, and family caregivers’ preferences play a critical role [1, 3, 4, 9, 16, 17, 18]. The issue of whether or not preferences change over time as illness progresses is important. There are indications that some people change preferences [1, 5, 8, 19], but evidence remains limited. Finally, there are people who do not have a clear preference, who prefer places other than home [1, 4, 5], and for whom other places may still be compatible with a ‘good death’ [3, 9, 10, 20].

Despite established evidence, there are significant gaps in understanding the diversity in preferences for places of EOL care and death (we refer to both as 'dying places'), and how these unfold as people approach death. Critically, the majority of studies have focused on the experience of cancer in high‐income countries. Most of the existing research is retrospective or cross‐sectional, relies on proxy report, and focuses on the endpoint (place of death) rather than what precedes and determines it (places of care). To provide patient‐centred care in the preferred place, we need to better understand people's lived experiences and preferences in different parts of the world with diverse socio‐cultural norms and healthcare systems. Hence, we aimed to explore how preferred and actual dying places unfold for adults with a life‐threatening illness and their family caregivers (including non‐relatives) in the Netherlands, Portugal, Uganda, and the United States. To facilitate cross‐country contextualisation, we include a text box with information on healthcare systems and key characteristics on dying places of the four countries (Box 1) [6].

Box 1. **Contextual background of the four countries**

*This description is reproduced from ‘Places of end‐of‐life care and death in health policies of four countries (EOLinPLACE)’ Cited from van de Beek SH, Gomes B, Eckels K, Pinto S, Sanguedo B, Olet DA, et al. Places of end‐of‐life care and death in health policies of four countries (EOLinPLACE Project). Health & Place. 2025;96:103534*.

Netherlands: The Dutch healthcare system is classified as an Etatist Social Health Insurance System [21]. All residents are required to have basic insurance from competitive, private, not‐for‐profit insurers under government regulation. Premiums are independent of income with a healthcare benefit to support people with lower incomes [22]. The main causes of death are ischaemic heart disease, cancer and dementia (2019) [22]. There is a strong preference for home death (84%; 2023) [23] and nursing homes are the most common least preferred places to die (42%; 2010) [24]. However, only 34% (2020‐21) died at home [25] and 23% (2023) in the hospital [59]. 34% of people with palliative care needs die in care homes (2022) [26].

Portugal: The Portuguese healthcare system is classified as a tax‐funded National Health Service system, providing universal and extensive coverage with some out‐of‐pocket payments. Care is mostly provided by public healthcare facilities (government) [21]. The main causes of death are ischaemic heart disease and stroke (2019) [22]. Most people prefer to die at home (51%) [11, 23] and the most common least preferred place to die is hospital (29%) [24]. The majority dies in a hospital (63%) [11] and only 23% (2020‐21) died at home [25].

Uganda: The Ugandan healthcare system has private, not‐for‐profit, faith‐based and public government funded facilities, and delivers decentralised health services. Private companies invest in hospitals, clinics, and pharmacies. There is no national health insurance coverage, but there is private health insurance provided by insurance companies. Some hospitals run their own health insurance schemes [27, 28]. The main causes of death are communicable diseases like tuberculosis and HIV/AIDS (2019) [22]. While dying at home is not found important by all patients (only 25%) [29], most people prefer to die at home (70%; 2000) [30]. The most common place of death is home (48%) and 35% die in health institutions (2020‐21) [25].

US: The US healthcare system is classified as a Private Health System, market‐driven and dominated by private insurers and private, for‐profit healthcare providers [21]. Financing is dependent upon private insurance, deductibles, out‐of‐pocket payments and federal programmes like ‘Medicare’ and ‘Medicaid’ [21, 31, 32]. The main causes of death are ischaemic heart disease and dementia (2019) [22]. While many people prefer to die at home (86%; 2004) [33], only 34% do (2020‐21) [25]. The other large portion dies in hospitals (36%) and hospice‐ and LTC facilities (27%; 2018) [11, 34].

## 2 Methods

### 2.1 Study Design

As part of a systematic comparative ethnography of the EOLinPLACE Project [35, 36], we (international and interdisciplinary team) conducted a cross‐national, qualitative, longitudinal study in different socio‐cultural settings. Between June 2023 and August 2025, we captured (adult and paediatric) patient and family caregiver pathways regarding preferred and actual dying places over time. The study reported in this paper only focuses on the adult patient pathways. Guided by a hermeneutic orientation [37, 38], we combined a constructivist interpretivist approach to explore personal narratives [39, 40], with a critical realist emphasis on underlying structures that may shape those narratives (e.g., healthcare access, cultural norms) [38], relevant to our international comparison. We assumed, based on prior evidence, that perceptions and experiences in the last phase of life can be dynamic, shaped by personal circumstances, social interactions, care context and cultural background. A pragmatic approach to thematic analysis [41], with a delineated codebook, allowed for an in‐depth exploration of internal perspectives and a cross‐national comparison. Methods and results were reported using the COnsolidated criteria for REporting Qualitative research (COREQ) checklist (Appendix A) [42].

### 2.2 Study Setting

The study setting was multi‐sited [36], as we aimed to closely follow patients facing a life‐threatening disease and their family caregivers in each country. Participants were recruited through hospital care staff (physicians and nurses from palliative care teams or specific departments treating patients with life threatening illness e.g., oncology department) identifying the eligible patients in our recruitment sites: an academic hospital and a regional centre for rehabilitation in the western urbanised part of the Netherlands; an academic integrated healthcare provider in the central, urbanised region of Portugal; an academic public hospital in an urbanised region of Uganda; an academic hospital located in an urban region of the Midwestern US; or through self‐referral (information leaflets in waiting rooms and online recruitment, in the Netherlands). Recruitment decisions were made progressively, as the study design required sensitivity, flexibility and individual assessment.

### 2.3 Study Population

In total, 35 eligible patients (≥ 18 years) with a life‐threatening disease in advanced stage (cancer, dementia, heart or cerebrovascular and neuromuscular diseases) were purposively sampled and approached [36]. Of these, 14 patients (cancer n = 6; dementia n = 2; heart and cerebrovascular disease n = 3; neuromuscular disease n = 3) and their family caregiver(s) (n = 18) were included and followed for 3 weeks to 18 months, either until death or until the study concluded. Others (21/35) did not join the study due to non‐response, faster‐than‐expected decline or death, burden of follow‐up interviews, or feeling overwhelmed by the illness or by professional interactions. Exact numbers of individuals approached cannot be determined, e.g., due to self‐referral strategies such as the online recruitment in the Netherlands. Because conditions are not mutually exclusive, participating patients were grouped according to the diagnosis that prompted their inclusion in our study. Patients with cognitive impairment with a wish to participate were interviewed, with consent of their legal representative. Patients were excluded if they had a life expectancy of more than 6 months to a year or less than 2 weeks; were too ill; were too overwhelmed by their situation; or did not understand the local language. Patients identified one or more family caregivers to join the study. Family caregivers were excluded if they were too overwhelmed or unable to understand the local language.

### 2.4 Data Collection

Multiple methods were used to obtain data relevant to answer the study objectives. The team consisted of a multidisciplinary team of researchers (details in Appendix C) with knowledge about palliative care, the country‐specific health systems and qualitative methods. Given the diversity in clinical, cultural and disciplinary backgrounds, we acknowledged that researchers’ perspectives could shape both data collection and interpretation (details in Appendix B). Trained, local fieldworkers (DO, IDS, KE, MD, SB ‐ details in Appendix C) with senior local supervision (BG, DT, ENG, JB – details in Appendix C) conducted semi‐structured interviews with patients and their family caregivers at regular intervals (at inclusion, middle and towards the end). Family caregivers were also interviewed after a patient's death to gain insight into the circumstances at the place of death. Interviews were planned according to relevant changes in preferred and/or actual dying places or significant decline in health. The interviews were conducted in an exploratory manner but included pre‐defined questions to capture key dimensions over time and allow for comparison across cases and sites (topic guides in Appendix D). Interviews with patients and caregivers were conducted separately to ensure individual privacy, or together when joint interviews were preferred. Patients with cognitive impairment were interviewed when possible but the majority of the information was acquired through proxy reporting by the family caregiver(s). When feasible, interviews were conducted at the patients’ places of care to facilitate observation or at the recruitment site (i.e., hospital). Alternatively, interviews were held online or by telephone when preferred by the participant or in case of long‐distances. Interviews were recorded, transcribed and pseudonymised in local language. Fieldworkers’ interview experiences and observations were documented in fieldnotes.

To capture relevant changes to participants’ pathways at the EOL, the fieldworkers had regular telephone contact with participants. Additional communication was maintained beyond the interview setting to check on status of change. The fieldworkers recorded the content of these conversations, their own interview experiences and initial reflections in their fieldnotes. The frequency of contact, timing of interviews and duration of follow‐up varied according to country‐specific adaptations, the course of events and preference of participants. Missed events and developments (e.g., transitions) were reconstructed by talking to the patient or family caregiver whenever circumstances permitted.

### 2.5 Analysis

The analysis was informed by principles of applied qualitative ethnography [41], allowing to combine methods, collect and manage data systematically, and execute team‐based and context‐sensitive fieldwork. This enabled a pragmatic approach, aligned with the pre‐defined research objectives. We combined principles of applied thematic analysis with principles of thematic network analysis [43, 44]. Applied thematic analysis is a systematic yet flexible approach, grounded in methodological rigour and transparency but not tied to a specific theoretical framework, making it well‐suited for applied and cross‐national research [43]. Thematic network analysis is a structured method for organising and interpreting qualitative data through a network of themes, providing a visual and systematic framework to move to higher‐level interpretations [44].

Local fieldworkers (BS, DO, IDS, KE, MD, SB) read and re‐read the transcripts and coded them using ATLAS.ti 25.0.1. There were a total of 46 transcripts accounting for around 1955 min of interviewing and summaries of 4 interviews, reconstructed by fieldworkers due to audio recording problems. To preserve cultural and linguistic nuances of the data, transcripts were maintained in the local language, applying codes in the common language of English. We adopted an eclectic approach, remaining open to different coding methods and deciding during data collection and coding which method would likely yield a substantive analysis [45]. We used a delineated codebook with elemental coding methods (i.e., basic, focused filters for coding), assigning descriptive (i.e., labels to summarise the topic of an excerpt) and structural (i.e., content‐based labels relating to the objectives and categorising the data) codes [45]. Additionally, we allowed for simultaneous coding (i.e., two or more codes assigned to the same excerpt) and sub‐codes (i.e., a second‐order coding strategy that enriches primary codes and supports nuanced categorisation) [45]. In the process of developing the codebook, existing codes were revised and inductive codes were added, grouped or removed. Across countries, the codebook was pilot tested and refined until consensus was reached (codebook in Appendix E). Fieldworkers met both online and in person to reflect on the coding process and discuss preliminary themes. Within each country, randomly picked transcripts were double‐coded by other coders (either native fieldworkers or local supervisors). After coding, Portuguese and Ugandan transcripts were translated into English to facilitate further in‐depth analysis led by SB (Dutch transcripts were analysed in the original language). Before engaging in deeper analysis of the coded transcripts, concise summaries of the participant stories were developed, and participant pathways were visually mapped. By further sub‐coding, (re‐)grouping, and connecting codes to preliminary themes, we explored the level to which themes were substantiated by the data. Fieldnotes were used to substantiate the analytic process. In the results, we have indicated which (sub‐)themes were more salient in a certain country. Given the differences in culture and approaches to EOL care (including palliative care), rather than emphasising differences we decided highlighting commonalities and diversity, irrespective of country or patient group, would be more meaningful. More details on the methods are included in Appendix B.

### 2.6 Ethical Considerations

Ethics committees of the host, recruiting and national organisations as applicable approved the research – Faculty of Medicine of the University of Coimbra (068‐CE‐2022), Coimbra Health – Integrated Delivery System (OBS. SF.085‐2022), Mulago National Referral Hospital (MHREC 2022‐70), Uganda National Council of Science and Technology (SS1537ES), Leiden University Medical Centre (non‐WMO division 3, nr 22‐3074) and University of Kansas Medical Center (STUDY00150249). We adhered to the ethical principles for medical research involving human subjects as described in the Declaration of Helsinki [46]. Reasons for doing the study were explained to eligible participants. Informed consent was obtained from all participants; for patients with cognitive impairment informed consent was obtained from their legal representative. Interview transcripts were pseudonymised and stored in a safe location. We followed a distress protocol to recognise and act on signs of distress in participants caused by or exhibited during the study [36]. Personal and medical needs of patients and family caregivers took priority over data collection.

## 3 Results

### 3.1 Participant Characteristics

In total, 8 of 14 patients died during the study period. Place of death was recorded for all who died except one, from the US (we were unable to contact the family caregiver after death). Table 1 shows further participant characteristics.

**Table 1:** Participant characteristics.

*Participant No*.	*Country code* 1	*Gender*		*Age groups* (yrs)		*Diagnosis of the patient* *	*Relationship between patient & family caregiver respectively*	*No. of interviews executed* 5			*Place of death* 2	*Bereavement interview* 3
		*Patient*	*Family caregiver*	*Patient*	*Family caregiver*			*Patient*	*Family caregiver*	*Joint*		
**01**	NL	Female	Male	60‐69	60‐69	Cancer	Married	3	1	0	Alive at end of study	N/A
**02**	NL	Female	Male	50‐59	60‐69	Neuromuscular disease	Married	0	0	3	Alive at end of study	N/A
**03**	NL	Male	Female	70‐79	70‐79	Neuromuscular disease	Married	0	0	4	Alive at end of study	N/A
**04**	NL	Male	Female	90‐99	60‐69	Dementia	Father & daughter	0	0	2	Alive at end of study	N/A
**05**	PT	Female	Male	50‐59	50‐59	Cancer	Married	0	1	1	NH	Yes
**06**	PT	Female	Male	60‐69	60‐69	Cancer	Married	3	1	0	PCU	No
**07** +	PT	Male	Female	70‐79	30‐39	Heart or cerebrovascular disease	Father & daughter	0	3	2	PCU	Yes
**08** +	UG	Female	Male	70‐79	20‐29	Heart or cerebrovascular disease	Grandmother & Grandson	0	4	1	Hospital	Yes
**09** +	UG	Male	Male	60‐69	30‐39	Cancer	Father & son	0	3	1	Home	Yes
**10**	UG	Female	Female	70‐79	40‐49	Dementia	Aunt & niece	0	3	0	Home	Yes
**11** +	UG	Male	Male	30‐39	40‐49	Heart or cerebrovascular disease	Friends	0	4	1	Alive at end of study	N/A
**12**	US	Female	Female	70‐79	—	Cancer	Aunt & niece	2	0	1	Unknown^4^	No^4^
**13**	US	Male	Female	40‐49	—	Neuromuscular disease	Life partners	0	0	3	Alive at end of study	N/A
**14**	US	Female	Male	60‐69	—	Cancer	Life partners	2	1	0	Hospital	Yes

* Diagnosis that prompted inclusion in the study; some participants were diagnosed with multiple chronic, life‐threatening diseases.

^1^ Country codes are NL (the Netherlands), PT (Portugal), UG (Uganda), and US (the United States).

^2^ Places of death abbreviations: NH (nursing home), and PCU (palliative care unit).

^3^ N/A: not applicable.

^4^ After this patient died, the family caregiver was lost to follow‐up so place of death is unknown and a bereavement interview could not be executed.

^5^ Includes n = 4 interviews that were not recorded or transcribed because of technical issues. These interviews were reconstructed as detailed summaries in fieldnotes and incorporated in the analysis.

^+^ For some patients, multiple family caregivers were identified. The table shows the primary contact included. Additional caregivers were the son of patient 7, the son of patient 8, the wife of patient 9, the father for patient 11.

### 3.2 Preferences for Dying Places

Across countries, most participants (patients and family caregivers) preferred home as dying place. Main reasons behind this desire to be home were maintaining everyday routines, the familiar rhythm of daily life, a sense of comfort, adhering to the patient's wish, and being around loved ones. Most prevalent reasons to prefer care settings other than home (e.g., hospice, palliative care unit, hospital, nursing home) were the level of dependency (e.g., feeling like a burden), safety, family caregiver burden, desire for the patient to gain social contact, (access to) treatment, and symptom management. If home was not preferred as the place of death, it was for similar reasons as previously stated, with the additional concern of avoiding anticipated trauma of death at home for family caregivers. Some people were more comfortable stating places they did not prefer, rejecting a place rather than choosing one as preferred. While most participants were able to articulate their preferences, some had difficulties clearly outlining their preference, either because they wanted to avoid thinking about it or because they felt their care pathway depended on other factors (e.g., future care needs).

### 3.3 (Non)Alignment between Preferred and Actual Dying Places

All patients were, at one point, able to receive EOL care at their preferred place. If a patient transitioned to a place, most often from home to a healthcare facility, it was sometimes because patients or family caregivers felt a need to seek higher‐level care. Most transitions, even when unplanned, reflected what patients wanted at that moment. Reasons why actual care pathways unfolded as they did included healthcare system structure, geographical disparities, (not) having skills to navigate the healthcare system, socio‐economic positioning, practical logistics (e.g., transportation means), and physical (in‐)dependency.

Of the eight patients who died during the study period, three died at their preferred place of death (home or palliative care unit). For the three patients who died in a hospital or nursing home (Table 1), reasons for non‐alignment between preferred and actual place of death were critical medical situations (heart attack and breathlessness), excessive family caregiver burden, or anticipated trauma of death at home for the family caregiver. For two patients, (non‐)alignment between preferred and actual place of death could not be determined, as one patient was unable to articulate preferences due to unconsciousness (proxy‐reported preference was home, and death occurred at home) and one patient died in a place unknown to the fieldworkers, due to loss to follow‐up with the family caregiver. To support the narrative results, illustrative patient pathways including the dying places were made (Appendix F).

In the rich and complex data there were recurring themes that appeared important for participants, highlighting more fundamental, underlying conditions influencing preferred and actual dying places [1]: Beyond the preferred: choosing otherwise [2]; Family caregiver commitment and burden affecting realisation of patient preferences; and [3] Navigating care shapes dying places.

### 3.4 Themes

#### 3.4.1 Beyond the Preferred: Choosing Otherwise

This theme examines how considerations such as the burden of receiving care, anticipated trauma of death at home for family caregivers, and addressing urgent care needs, shape participants’ preferences regarding dying places, creating a tension between what is genuinely preferred and the choices made in response to these influences (whether well‐considered or impromptu).

While among the Ugandan patients in our study the issue of feeling like a burden to family caregivers was not explicitly stated, patients in other countries reflected on the potential burden their care might place on their family caregivers when remaining home, contributing to feelings of being a burden. This concern manifested in two ways. For some, it was grounded in practical realities such as the family caregiver's ability to continue to provide support or intensify care in the future. For others it was connected to personal values, grounded in a desire to maintain independence.

Patient: ‘If I could, I would stay at home till I die.’

Interviewer: ‘So you are kind of thinking you might need more care than your partner could give you and so…’

Patient: ‘Yeah, he's sick too [progressive illness]. […] I don't want to go away. […] At the same time, I'm killing him [partner], you know?’

(Patient 14; US)

Additionally, the ‘anticipated trauma of death at home for family caregivers’ influenced choice for dying places. This was salient in interviews with Dutch and Portuguese participants, but not with Ugandan and US participants. While place of care was preferred to be home for many reasons, the anticipated emotional weight of experiencing a death at home for family caregivers played an important role in choosing a place of death other than home. This was expressed as a concern by both patients and family caregivers.

I didn't want that [partner dying at home] here. If the trauma was already big, then it would be much worse.

(Family caregiver patient 5; PT)

Finally, we found urgent care needs may influence choice for a place other than home. Participants from our study shared how the urgent need for higher‐level care that could not be provided in the place they were (home), informed a decision to transition to a healthcare facility (most often the hospital). These transitions did not reflect a patient or family caregiver's (shifting) preference for healthcare facilities as place of care. It was the available care that influenced choice for that place, rather than a shift in preference. Transitions in response to urgent care needs appeared to be shaped by multiple considerations, like the need for symptom management, either following standard urgent care procedures (i.e., hospital admission) or guided by patients’ or family caregivers’ judgement that higher‐level care was required. Ultimate motivations were to achieve comfort, postpone death, improve health, or prolong life. These transitions were however considered acceptable only in moments of need. Once the need subsided, some participants expressed a strong wish to return home, highlighting concerns such as the risk of infection.

Automatically she would be at her home but if her condition worsens and (she) needs hospitalisation, we would take her back to hospital.

(Family caregiver patient 10; UG)

#### 3.4.2 Family Caregiver Commitment and Burden Affecting Realisation of Patient Preferences

This theme explores how family caregivers and their dynamics with patients can influence dying places, including whether patient preferences are realised. We found that when patient and family caregiver preferences aligned, this led to a shared idea about dying places and a collaborative effort to achieve preferences, when possible. Yet, when tensions arose, these dynamics became more complex. There was a range of considerations reported by family caregivers, including respecting the patient's wish, a sense of obligation to care for or protect the patient, and the capacity to (continue) providing care.

When family caregivers were asked about their preferred place of death for the patient and the underlying reasons, a common response was their desire to honour the patient's wish. In some cases, family caregivers repeatedly emphasised that it was ‘the patient's choice’, making it difficult for the interviewer to discern their own preference. Even when family caregiver burden was overwhelming and family caregivers expressed a personal preference for institutional care, they sometimes refrained from acting on it, because the patient preferred to stay at home. In other cases, family caregivers chose to provide care in the patient's preferred place out of a sense of obligation. This was grounded in a desire to reciprocate the care and support they had previously received from the patient, or the expectation that the patient would have provided similar care if roles were reversed.

In principle, once I sink my teeth into something, I don't let go. […] She always said from the very beginning: “[…] I want to stay at home”. So we're going to do everything we can to make that happen.

(Family caregiver patient 2; NL)

I have to do so. […] she was my mother who looked after me from the age of 6 months. She was my aunt, but she was also my mom.

(Family caregiver patient 10; UG)

In some cases, the needs and ability of family caregivers influenced actual dying places in a way that patient preference could not be met. When the caregiver burden became unmanageable to family caregivers due to their own health issues, being overwhelmed, or insufficient formal home care support, it led to a transition to another place than home. When, in contrast, family caregivers had strong informal and formal support networks or prior (professional) experience with caregiving, this facilitated staying at home. In some cases both the patient and the family caregiver acknowledged staying at home might not be feasible. Anticipating the family caregiver's inability to sustain care, alternative plans were made and accepted. However, this did not necessarily reflect preference.

And she accepted it [going to the nursing home]. It was the last thing she wanted. She wanted to stay. To try and die here [home]. Her safe place was to die at home. I was the other way around. I didn't want that [death at home] here. […] What I did in the end was only to give her a little more stability. Get her in a better place [nursing home].

(Family caregiver patient 5; PT)

#### 3.4.3 Navigating Care Shapes Dying Places

How people navigated care and how that influenced dying places was the third theme in our data. This theme explores the influence of the healthcare system structure and care options across countries and how patients’ and family caregivers’ knowledge and skills influence the ability to navigate the healthcare system.

Access to healthcare varied widely within and across countries. There were differences in access to home care and accessing care in healthcare facilities. Barriers and facilitators were related to pressure on the healthcare system (e.g., waiting lists), geographical disparities, financial and social (dis‐)positioning, and family resources. What the system offered, and thus whether individuals had options, varied considerably. For instance, in the US, healthcare and insurance packages influenced where patients could receive care, shaping EOL options and treatment decisions. Hospice care does not include 24/7 home care, so remaining at home was not fully facilitated by the system, whereas life‐prolonging treatment (e.g., a trachea cannula in neuromuscular disease) facilitated continuous care options, enabling a patient to remain at home. Additionally, the diagnosis and the illness progression influenced whether individuals were able to access appropriate care in a timely manner.

… the fact that we were very far from the hospital, sometimes even transporting her to the hospital was hard due to the limited transport means.

(Family caregiver patient 8; UG)

We had started talking about the trach [trachea cannula]. Didn't have it actually set yet. He got pneumonia and had to have it. I mean, it was either have the trach or go home on hospice, basically at that point. […] I quit my job, so I could care for him fulltime. But the only way I could do that is if I was getting paid to take care of him, to replace my salary. So, I get paid through Medicaid, from the State.

(Family caregiver patient 13; US)

Knowledge and skills related to understanding the illness and navigating the healthcare system (often called death literacy) also played a role in shaping participants’ preferred and actual dying places. Across the data, this showed in various ways, including the time it took to reach diagnosis, the extent to which the diagnosis and prognosis were understood, and the ability to recognise and make sense of disease severity. Participants’ awareness of EOL care and their understanding of what could be expected from palliative care teams also influenced how they navigated the system and anticipated future needs. Cognitive impairment that could be due to dementia added further complexity. Moreover, participants’ familiarity with the healthcare system structure and the available care settings, advanced educational background (which may facilitate understanding and acquisition of knowledge), or prior exposure to healthcare (e.g., professional experience) shaped their ability to articulate informed preferences. Participants with less familiarity were unaware of all available care settings. In this way, knowledge and skills not only shaped opportunities for choice, but also enabled people to develop a preference, grounded in a better understanding of their range of options.

[Interviewer asks where the caregiver would like her father to be cared for]

*Caregiver:* ‘Better at the hospital. […] I also don't know any other options besides that one’

*Interviewer:* ‘A Palliative Care unit, or a Continuous Care one…’

*Caregiver:* ‘Yes, that. In the hospital, in a unit like one of those, yes.’

[Interviewer explains that in the region where they live, these units are not inside their hospital of reference].

*Caregiver:* ‘Without knowing it, I can't, right? I can only speak of what I know.’

(Family caregiver patient 7; PT)

##### 3.4.3.1 Interactions Between the Three Themes

**Figure 1:**
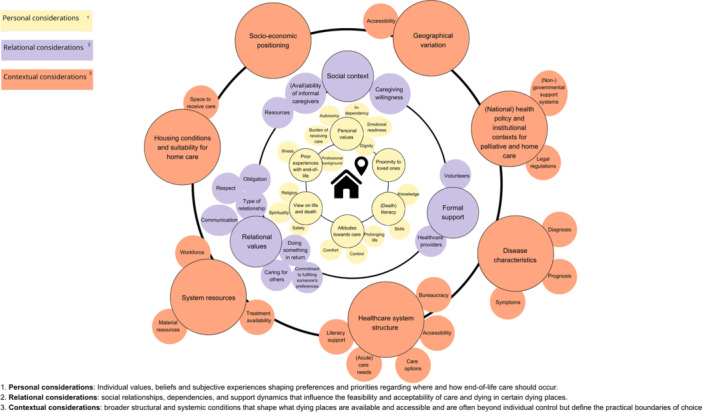
Personal, relational, and contextual considerations shaping preferred and actual dying places.

The study data revealed a complex interaction between the multiple considerations that influenced preferred and actual dying places. In Figure 1 these considerations are mapped into patient‐related (e.g., personal values, care needs), relational (e.g., family caregiver (avail)ability, anticipated burden), and contextual (e.g., healthcare system structure) considerations. The figure highlights the multi‐layered nature of EOL decision‐making. When key considerations are not met, individuals may be prompted to choose or prefer an alternative place. Which considerations are most influential varies by person and situation and operates on a case‐by‐case basis, reflecting a dynamic negotiation in which preferences are constantly balanced against reality. Some considerations, such as urgent care needs, appear to be of shared importance for patients and family caregivers across the four countries in informing transitions between places.

## 4 Discussion

In this study we captured EOL pathways, unfolding preferences around dying places (i.e., places of EOL care and death) of adults with a life‐threatening illness and their family caregiver(s) in the Netherlands, Portugal, Uganda, and the US. Preference for home as a dying place was common although not universal. Our findings show that personal, relational, and contextual considerations informed decisions for dying places, suggesting that preferences are the result of weighing and prioritisation. Preference, in this sense, emerged as an ethical and situated view: not just the consideration of an earlier wish, but a response to what matters most in a changing reality. Participants associated home with autonomy, dignity, proximity to loved ones, and doing something meaningful (as family caregiver) while preferences for other settings related to concerns about being a burden to others, anticipated trauma of death at home for family caregivers, the need for care and symptom management, informal support and family caregiver burden, the (ability to navigate the) healthcare system structure, and formal care limitations (e.g., homecare intensity).

### 4.1 Considerations for Preferences

Our findings naturally reflect the multilevel patterns described in socioecological models [47, 48]. Participant narratives show how the interplay of considerations interact to shape EOL decisions, which aligns closely with prior work and supports a multilevel lens to EOL decision making [2, 9, 14, 17]. Preference may be dependent on these considerations and can inform choice for another place [19]. The considerations identified in our study are striking in view of our cross‐cultural international project. Even though the prominence of these considerations may vary by country, their interaction and the way they influence preference, choice and the actual course of where death happens, seems to be a shared phenomenon. Nevertheless, not all considerations were salient across patients and countries which may be attributed to the variety in socio‐cultural contexts.

### 4.2 Family Caregivers

Family caregivers are active participants in shaping the outcome of patient preferences, and thus could likely enable or set limits on what is achievable [4, 49, 50]. Based on our findings we postulate that it is not just the family caregiver's preference but the underlying concerns (such as caregiver burden) informing the preference that shape choice for dying places. Literature highlights how experiencing a death at home can affect a family caregiver's perception and meaning of home [51]. Notwithstanding, we also observed family caregivers’ commitment to achieve a patient's preference for home which may stem from relational values such as respect, obligation, solidarity, and doing something meaningful, aligned with findings elsewhere [1, 52]. The family caregiver thus could be a decisive factor determining whether home as dying place is feasible. This was also reflected in our study, where, despite differences in collectivist and individualist norms, the patient and family caregiver interaction was important across all four countries. Recognising that preferences and decisions within the patient‐family caregiver dynamic are often a matter of negotiation is essential [9, 53] and emphasises the importance of addressing these relational processes and facilitating constructive dialogue to negotiate potentially opposing needs and preferences [54]. Including family caregivers in the decision making process concerning preferences in EOL care and taking notice of their needs is critical given their pivotal role as facilitators for patient preferences [4, 55]. Further exploration of drivers underlying family caregiver preferences may yield a deeper understanding of what truly shapes where people die, allowing for more actionable insight to achieve informed, person‐centred, EOL care decision‐making.

### 4.3 Place As a Construct

Previous research indicates that the way people consider dying places is complex and layered [1, 2, 3, 56, 57, 58], with some studies suggesting that place is not an overarching preference or priority at the EOL [3, 57]. Our findings show that both preferred and actual dying places are shaped by a response to personal, relational and contextual considerations, suggesting that preferences for place are informed by, and maybe even subordinate to, context. Aligned with findings elsewhere, we propose that decisions about actual dying places are not merely individual choices but relational and ethical negotiations, influenced by responsibilities to others, availability of informal care, institutional rules, socio‐economic resources, and proximity to health services [9]. We suggest that, instead of interpreting place as being a second‐order preference to other EOL priorities, it may be more valuable to view place as encompassing the personal, relational, and contextual considerations that shape it and give it meaning. In this sense, ‘place’ is not merely a physical location but a construct emerging from a dynamic balancing of multiple EOL priorities [20]. When the necessary conditions for care in a certain place are no longer met, a move to another setting could thus be understood as a shortcoming (of healthcare provision) to provide choice, or as a reasoned adjustment of a variety of priorities; not necessarily a direct conflict with preferences but rather a choice in response to reality [17, 59]. Accordingly, discussions about preferred place must address the conditions crucial to a good quality of dying for that person and a good quality of life for their family caregivers, which extends into bereavement. Further research into place as a construct, particularly ‘home’ (e.g., “at‐homeness” and feeling at home), could increase our understanding of how these factors shape preferences and choices regarding dying places and help to align with core needs of patients and family caregivers.

### 4.4 Place of Death As Quality Indicator

Preferred place of death and its alignment with actual circumstances is considered to be a quality indicator of EOL care [11]. While adhering to patient and family caregiver preferences is key to delivering person‐centred and responsive care [60], any use of place of death and its congruence with preference as a quality proxy must consider the complexity of how preferences are expressed, understood and acted upon. The way people talk about where they prefer to be at the EOL depends on how the question is framed and on what “place” means to them at that moment (physically, emotionally, and relationally). This underscores that preferences and decisions for dying places are deeply contextual and relational. If preference is an adaptive choice where people trade‐off, balancing benefits and risks of reality, continuous dialogue is needed. Therefore, the recording of an original preference and how that evolves into a possibly different outcome may in itself be a sign of person‐centred care and quality of EOL care.

To complicate matters further, home is often idealised as dying place and linked to a ‘good death’ [6, 9, 10]. While there is evidence that supports an association between dying at home and better outcomes (e.g., greater peace, lower grief complications) compared to hospital [61, 62, 63], our findings suggest that the feasibility and implications of being at home at the EOL and the pathway that precedes a home death are complex and dynamic. Importantly, our findings show that, even when contextual conditions for care at home are met, patients and family caregivers may still choose to be cared for or die in another setting due to other personal or relational considerations. In these cases, alternative locations may thus better support quality EOL care. Additionally, some of the defining attributes of ‘home’ (e.g., safety, company) may still be experienced in other settings [20]. Hence, we argue that dying in the preferred place is a more valid quality indicator than home death [54], which is often assumed to be universal in current health policies [6]. Also, focusing solely on place of death risks overlooking important aspects of the process preceding death that influences quality of life, care, and dying [64]. We put forward that quality may be assessed by the extent to which individuals are provided continuous choice, adapting to changing circumstances.

### 4.5 Strengths and Limitations

To our knowledge, this is one of few qualitative studies on EOL that draw on longitudinal data collection directly from the perspective of patients and family caregivers, conducted across four countries with contrasting socio‐cultural contexts. Despite challenges in recruitment, we were able to include patients with various life‐threatening illnesses in advanced stage, capturing experiences over time and across countries. This led to insights into the way patients and family caregivers in different parts of the world deal with the ever‐changing circumstances at the EOL. The fieldworkers’ presence and the topic of conversation may have influenced participants’ awareness and decision‐making, potentially shaping preferred and actual dying places. Participants thus may have considered EOL preferences more consciously than people who did not participate, which could be considered a strength as greater awareness and knowledge support informed decision‐making processes.

Cross‐national qualitative research offers valuable insights into complex phenomena across diverse cultural settings and healthcare contexts, yet its methodological challenges remain underreported. In line with existing literature, we encountered challenges inherent to conducting longitudinal, cross‐national, qualitative research, including conceptual alignment, linguistic differences, translation of transcripts, bridging team diversity (disciplines and cultures), and maintaining analytical consistency across countries [65, 66, 67]. Because of small sample sizes, limited saturation, variability in data across countries and reliance on participant narratives, we focussed on commonalities and diversity, providing insight into considerations occurring in EOL care around the world.

## 5 Conclusion

This study provides critical insight into how preferences for dying places are constructed, and how they develop over time across different regions in the world. These cross‐national findings show the importance of viewing place not merely as a physical location but as a construct emerging from a complex interplay and constant prioritisation of the personal, relational and contextual considerations that shape it and give it meaning. Although the prominence of the relevant considerations may vary by country, their interaction and the way they shape preferred and actual dying places appears to be a shared phenomenon. Understanding the considerations that matter most to patients and family caregivers and how these interact, may help to better accommodate preferences for dying places. Our findings underscore how alignment between preferred and actual place of death is a fundamentally important yet complex phenomenon, influenced by changing circumstances (e.g., place of care) and by when and how preferences are discussed. In future research, measures of congruence between preferred and actual place of death should account for the dynamic nature of preferences and their underlying considerations. Better understanding of the underlying needs that shape these preferences may help inform both policy and practice with the goal to achieve preferred dying places and align EOL experiences more closely with core patient and family caregiver values. Clearly, at the EOL ongoing dialogue about what people want and where they want to be is enormously important, in view of the fluidity of various EOL priorities and considerations. Further research from other parts of the world is needed to better understand the role of preferences and needs of patients and family caregivers in relation to dying places, to ensure person‐centred EOL care that is responsive to differing social, cultural and healthcare contexts.

## Author Contributions

**Sifra Hannah van de Beek:** conceptualisation, data curation, formal analysis, investigation, methodology, project administration, validation, visualisation, writing – original draft, writing – review and editing. **Krista Ann Eckels:** conceptualisation, data curation, formal analysis, investigation, methodology, validation, writing – review and editing. **Dorothy Adong Olet:** conceptualisation, investigation, methodology, validation, formal analysis, data curation, writing – review and editing. **Inês Dias da Silva:** conceptualisation, investigation, methodology, validation, writing – review and editing, formal analysis, data curation, project administration. **Mayra Delalibera:** conceptualisation, investigation, methodology, validation, formal analysis, data curation, writing – review and editing. **Joanna Veazey Brooks:** conceptualisation, methodology, project administration, resources, supervision, validation, writing – review and editing. **Elizabeth Namukwaya:** writing – review and editing, conceptualisation, methodology, validation, project administration, supervision, resources. **Jenny Theodora van der Steen:** conceptualisation, methodology, validation, writing – review and editing, project administration, supervision, resources. **Yvette Milene van der Linden:** resources, supervision, writing – review and editing, validation. **Rui Garcia:** validation, resources, writing – review and editing. **Barbara Gomes:** writing – review and editing, conceptualisation, data curation, funding acquisition, methodology, project administration, supervision, resources, validation, writing – original draft. **Dorothea Petra Touwen:** writing – original draft, writing – review and editing, conceptualisation, data curation, project administration, resources, supervision, methodology, validation, visualisation.

## Ethics Statement

Ethics committees of the host, recruiting and national organisations as applicable approved the research – Faculty of Medicine of the University of Coimbra (068‐CE‐2022), Coimbra Health – Integrated Delivery System (OBS. SF.085‐2022), Mulago National Referral Hospital (MHREC 2022‐70), Uganda National Council of Science and Technology (SS1537ES), Leiden University Medical Center (non‐WMO division 3, nr 22‐3074) and University of Kansas Medical Center (STUDY00150249). We adhered to the ethical principles for medical research involving human subjects as described in the Declaration of Helsinki. Reasons for doing the study were explained to eligible participants. Informed consent was obtained from all participants; for patients with cognitive impairment expressing a wish to participate, informed consent was obtained from their legal representative.

## Conflicts of Interest

The funder had no role in the design and conduct of the study, and decision to submit the manuscript for publication. Jenny van der Steen reports financial support provided by European Research Council and she co‐chairs the Interdem task force End of Life and Palliative Care. Barbara Gomes is scientific advisor of the La Caixa Foundation Programme of Comprehensive Care for People with Advanced Diseases in Portugal since 2018.

## Supporting information

Supporting File 1

Supporting File 2

Supporting File 3

Supporting File 4

Supporting File 5

Supporting File 6

## Data Availability

Data cannot be shared to protect study participant privacy.
