# Supplementary material for: How Preferences and Reality on Where We Die Unfold: A Four‐Country Longitudinal Qualitative Study (EOLinPLACE)
Source: Health Expect. 2026 Jul 3;29(4):e70732. doi: 10.1111/hex.70732 (PMC13332329; doi:10.1111/hex.70732)
Supplement: Supplementary file 1 — Supporting File 1 [file HEX-29-e70732-s001.docx]

**Appendix A: COREQ checklist**

**Article title**: How Preferences and Reality on Where We Die Unfold: A Four-Country Longitudinal Qualitative Study (EOLinPLACE)

| Item No. | Topic | Description | Page no. / Appendix |
| --- | --- | --- | --- |
| **Domain 1: Research team and reflexivity** | | | |
| 1. | Interviewer/ facilitator |  | 4 |
| 2. | Credentials |  | Appendix C |
| 3. | Occupation |  | Appendix C |
| 4. | Gender |  | Appendix C |
| 5. | Experience and Training |  | Appendix C |
| *Relationship with participants* | | | |
| 6. | Relationship established |  | Appendix B |
| 7. | Participant knowledge of interviewer |  | Appendix B |
| 8. | Interviewer characteristics |  | Appendix C |
| **Domain 2: Study design** | | | |
| *Theoretical framework* | | | |
| 9. | Methodological orientation and theory |  | 3-5 |
| *Participant selection* | | | |
| 10. | Sampling |  | 3 |
| 11. | Method of approach |  | 3 |
| 12. | Sample size |  | 3 |
| 13. | Non-participation |  | 3 |
| *Setting* | | | |
| 14. | Setting of data collection |  | 3 |
| 15. | Presence of non-participants |  | N/A |
| 16. | Description of sample |  | 3 & 6 |
| *Data collection* | | | |
| 17. | Interview guide |  | Appendix D |
| 18. | Repeat interviews |  | 3-4 |
| 19. | Audio/visual recording |  | 3-4 |
| 20. | Fieldnotes |  | 4-5 |
| 21. | Duration |  | 4 |
| 22. | Data saturation |  | 14 |
| 23. | Transcripts returned |  | N/A |
| **Domain 3: Data analysis and findings** | | | |
| *Data analysis* | | | |
| 24. | Number of data coders |  | 4 |
| 25. | Description of the coding tree |  | Appendix E |
| 26. | Derivation of themes |  | 4-5 |
| 27. | Software |  | 4 |
| 28. | Participant checking |  | N/A |
| *Reporting* | | | |
| 29. | Quotations presented |  | 7-10 |
| 30. | Data and findings consistent |  | 7-11 |
| 31. | Clarity of major themes |  | 7-11 |
| 32. | Clarity of minor themes |  | N/A |
