# Supplementary material for: How Preferences and Reality on Where We Die Unfold: A Four‐Country Longitudinal Qualitative Study (EOLinPLACE)
Source: Health Expect. 2026 Jul 3;29(4):e70732. doi: 10.1111/hex.70732 (PMC13332329; doi:10.1111/hex.70732)
Supplement: Supplementary file 2 — Supporting File 2 [file HEX-29-e70732-s003.docx]

**Appendix B: Methods**

**Article title**: How Preferences and Reality on Where We Die Unfold: A Four-Country Longitudinal Qualitative Study (EOLinPLACE)

*1. Study context*

Our study is part of a larger (EOLinPLACE) project comprising multiple studies aiming ‘to create a solid base for the first international classification to map preferred and actual places towards death grounded on what they mean to individuals (beyond a purely physical or medical view)’. The current study was designed to inform this classification, aiming to document trajectories of patients dealing with life threatening chronic illness, and explore the preferred and actual dying places. More information on the project’s aims can be found in the protocol paper.(1) In each participating country, one or more PhD students executed the research, supported and supervised by invited researchers, postdoctoral fellows, and senior investigators. Most team meetings were held online. Key discussions and brainstorming sessions about study results were conducted in person whenever possible.

*2. Research paradigm and reflexivity*

Our research question was aimed at understanding experiences and meanings around (changing) preferences for dying places and mapping actual pathways over time. Within our hermeneutic orientation, we constantly moved between understanding individual experiences (the data) and the broader context of the research settings. The constructivist interpretivist approach presupposes that knowledge is constructed through interactions between the researcher and participants, and realities are dependent on the people who hold them.(2, 3) Simultaneously, the critical realist perspective acknowledges the existence of underlying structures and mechanisms, such as healthcare systems and cultural norms that influence these realities; relevant to our international comparison. With this approach and being aware of our different socio-cultural and professional backgrounds, we continuously reflected on our pre-understandings. All fieldworkers reflected on their own positions, experiences, and potential biases throughout the research process. Amongst others, on:

- Their demographic characteristics, (cultural) background, upbringing and social position in society.
- Personal experiences and motivations for pursuing research in the palliative care domain, and how these experiences shape both personal and professional perspectives on end-of-life care.
- Awareness of prior work in palliative care, which may have shaped the data and analysis.
- Personal beliefs about home-based care versus other care settings as dying places.

To surface and manage these pre-understandings and potential biases, we employed several strategies. Reflections on positionality were noted in fieldnotes. Regular virtual and in-person team meetings included critical discussions of our pre-understandings and interpretations, allowing for reflexive consideration of how the researchers’ perspectives might influence the study, interviews, questions asked, the way rapport was established, and the way results were analysed (i.e., coding and theme development). Interestingly, for instance, the outsider perspective of SB on the coding of the other fieldworkers brought to light some pre-understandings or assumptions that could be challenged. These discussions enabled comparison of perspectives across countries and disciplines and helped to identify and challenge implicit assumptions. Through this process, we aimed to ensure that the analysis was not driven by a single cultural, clinical, or disciplinary perspective, but rather reflected a more nuanced, collectively constructed understanding of the data.

*3. Data collection*

*3.1 Recruitment*

Adult patients (≥ 18 years) with cancer, dementia, heart and cerebrovascular diseases, or neuromuscular disorders,(4) and a life expectancy of approximately 6 months were eligible. Eligibility was limited to these disease groups, in line with the focus of the EOLinPLACE Project.(1) Participants were purposefully sampled at clinical sites. Health professionals (physicians and nurses either specialised in palliative care or other specific diseases like lung diseases) approached patients who they considered not too ill or overwhelmed and who were sufficiently proficient in the local language, asking if they were willing to be contacted by the research team. They provided brief information about the study and/or shared an information leaflet. Patients and family caregivers either gave consent for the researcher to obtain their contact details or received the researcher’s contact details to reach out themselves.

When participants verbally indicated their interest in joining the study, the researcher reached out to the patient and family caregiver to provide more detailed information and – once agreed – acquire written informed consent in two copies (one for the participant and one for the researcher). Some participants opted out after this conversation for reasons such as an unexpectedly quick decline in health, a euthanasia appointment in the near future or being overwhelmed. Informed consent forms were securely stored, accessible only to the researchers working on this study in the respective country. For people with dementia (or their proxies) who wanted to join, the same conversations were held as for people without cognitive impairment. Proxies signed a different informed consent form and – when applicable – also signed the consent form of the person with dementia. Consent and assent were only accepted if the person with dementia was willing to join at that moment (to the extent that (s)he was able to clearly vocalize this).

The recruitment process varied across countries depending on local appropriateness, sometimes presenting unique challenges in one of the participating countries. In the US and Uganda, it was appropriate to compensate participants, whereas in the Netherlands and Portugal this was not common practice. In the Dutch setting, the recruitment strategy was expanded to allow for self-referral (i.e., online recruitment and information leaflets in waiting rooms at the clinical sites) and a broader inclusion window (≤1 year). Posts on calls for participation were shared on LinkedIn and through relevant palliative care organizations. In Portugal, to support recruitment, fieldworkers joined clinical team meetings to help identify eligible participants. Recruitment decisions were made progressively, as the study design required sensitivity, flexibility, and individual assessment.

*3.2 Interviewing*

All fieldworkers received online training from an advisory board member of the project, a representative of the International Alliance of Patients’ Organizations (IAPO). This training helped to ensure interviews with patients and family caregivers were conducted in a sensitive and appropriate manner. With this training, all fieldworkers were also trained to recognise distress in study participants and how they could apply the project’s distress protocol.(1) Participants could choose whether they wanted to have the interview conducted separately or jointly with their family caregiver. Participants with dementia were interviewed jointly, with their family caregiver who was also participating. This family caregiver both assisted in interpreting the patient’s responses to questions and responded to questions in their role as a caregiver. Only one of two participants with dementia was able to respond to questions in this study. He was asked the same questions as the other participants, and his answers were recorded. While answers might have derailed, off topic or not true (indicated by proxy), his story was informative as it clearly illustrated his level of comfort with his (preferred) place. We think it is extremely important to include the stories of people with dementia themselves. Hence, we included his story as such.

There was no relationship between fieldworkers and participants, prior to the study. During the study, sometimes a relationship developed in which personal interests and motivations for conducting the research were sometimes shared with participants when this arose naturally in the conversation.

Common challenges in data collection for cross-national qualitative studies were encountered such as team diversity (e.g., varying ways of prompting). The data (i.e., interview transcripts and fieldnotes) varied across countries. Differences in interview length and frequency, and the depth of questions and prompts used introduced variability within the data. To accommodate for this, fieldworkers captured contextual details, observations, and reflections that were not always evident in the transcripts of interviews in their fieldnotes. These notes included non-verbal cues, interactions, environmental factors, and initial interpretations. Additionally, during telephone interviews and other ways of contact for establishing rapport, fieldnotes were made.

*4. Analysis*

As this study was part of a large, cross-national project, developing the codebook was a collaborative effort. Each coder had in-depth knowledge of the healthcare system context of their respective country and research topic, ensuring culturally informed interpretations. Working as a team allowed for multiple perspectives in analysing and interpreting the data and through ongoing discussions, we ensured transparency. These exchanges often raised new analytical questions, informed the codes, and strengthened the overall analytical process. However, achieving coding consistency across countries proved complex, given the different levels of experience among fieldworkers, various educational and professional backgrounds, and the variability in linguistic and cultural contexts. To address these challenges pragmatically, we decided to develop a robust, delineated, and well-defined codebook, supported by procedures to ensure intercoder reliability. Additionally, we centralized the cross-national analysis with one fieldworker (SB), closely supervised by an experienced senior qualitative researcher (DT).

*4.1 Coding*

We made a robust and well-defined codebook by providing code definitions, when (not) to use a code and example quotes for when (not) to use codes. Changes were made according to discussions on code interpretations. Consistent with a reflexive approach, we did not calculate formal intercoder reliability; instead we repeatedly discussed how we understood and applied codes to develop shared understanding. In instances of discrepancies or disagreements, we discussed different code names that would help with applying the code consistently. The coding process was not perceived as a technical act. We acknowledge that coding is also a subjective interpretative process influenced by personal perspectives and predispositions, which is particularly important to consider when coding with multiple coders. Therefore, harmonizing our coding practices across teams was essential for ensuring analytic coherence and rigour. The coding process followed the framework and principles outlined by Saldaña (2021), whose typology of coding methods informed coding decisions.(5) We adopted a pragmatically eclectic approach, remaining open to different coding methods and deciding during data collection and coding which would be most appropriate and likely yield a substantive analysis.(5) The nature of our research question and objectives strongly influenced the coding decisions we made. These decisions were also shaped by the methodological requirements of the study: the longitudinal nature of the project and the diversity of data collected over time required a flexible yet structured coding approach.

Because our research question was primarily descriptive – focusing on exploring preferences, experiences, events and care trajectories – and because existing literature suggested that certain codes were likely to appear in our data, we started the coding process with a deductive codebook. Furthermore, these deductive codes were based on a pre-established coding system derived from a previous study and the interview topic guide, all while ensuring close alignment with the research aim – also known as structural coding.(5) We added codes inductively during the coding process. Additionally, we added the code ‘other’ to the codebook so we could flag quotes that needed a different code than available from the initial codebook; writing down in memo’s what new code we felt we needed.

To preserve the cultural and linguistic nuances of the data, all transcripts were coded in the original country-specific language, applying codes in English. Translation occurred only after all transcripts were coded with the iteratively tested, ‘final’ codebook, allowing SB to understand the excerpts to which codes were attributed during further analysis.

During the coding process, we had face-to-face team meetings, to discuss initial impressions of the data and raise analytical questions that could guide theme development (e.g., ‘*What surprised me?*’). These discussions served as a thematic reflection on the data and prompted critical discussions on why we were drawn to certain codes or pre-liminary themes. During these meetings, a number of coding decisions were made:

1. We agreed to code selectively, focusing only on segments of the data that were directly relevant to the research objectives (i.e., postmodern perspective on ethnographic texts).(5)
2. Contrary to the initial plan,(1) we decided not to code fieldnotes because, after revision, the fieldnotes did not yield substantial additional findings beyond the primary data sources (i.e., interview transcripts). Instead, they were used i) in team discussions, ii) to substantiate theme development, and iii) to reflect on our positionality in relation to the findings.
3. Primary coders had a colleague double-coding some transcripts to rigorously examine and audit the coding process. Additionally, SB reviewed coded material across countries to identify discrepancies and spark discussions on coding strategies that might be based on biases or pre-understandings.

We applied the grammatical coding methods of simultaneous coding (≥two codes assigned to the same excerpt) and sub-coding (a second-order coding strategy that enriches primary codes and supports more nuanced categorization).(5) Additionally, we used elemental coding methods, including descriptive coding (labels summarizing the basic topic of an excerpt, allowing for the categorization of data) and structural primary-codes (applying content-based phrases representing topics).(5) Finally, in our attempt to move toward deeper understanding, we applied emotion coding (labelling emotions recalled or experienced by participants) and value coding (labelling participant’s values, attitudes and beliefs).(5)

We re-coded portions of the data as we refined our methods and realized that some initial choices did not work because the codes were not aligned with the main research questions and therefore introduced noise into the analysis (e.g., too many value-oriented codes). This complicated analyses because these types of codes did not allow for consistent and rigorous application by all fieldworkers, and interpretations could vary too widely. Consequently, we excluded affective coding methods and focused on grammatical and elemental coding methods that directly addressed the research objectives, producing an organized inventory of the relevant data. Although descriptive coding offers limited theoretical depth, it facilitated the development of a coherent analytic framework for SB to continue with thematic analysis in alignment with our research aims.

*4.2 Theme development*

After the coding phase, which was a collaborative effort but conducted locally, the theme development was centralized. Before engaging in the deeper analysis of the coded transcripts, SB consulted each fieldworker individually to develop concise summaries of every patient and family caregiver story. By visually mapping, grouping, sub-coding and re-grouping, and connecting codes to preliminary themes discussed during in-person meetings with all fieldworkers, we explored which preliminary themes were truly substantiated by the data.

Analysis of the coded excerpts were based on principles of applied qualitative ethnography.(6) This method allows researchers to combine multiple methods, collect and manage data systematically, and execute team-based and context-sensitive fieldwork. Within this approach, we combined principles of Applied Thematic Analysis and Thematic Network Analysis. Applied Thematic Analysis combines inductive and deductive coding, emphasizes team-based analysis and intercoder reliability, and seeks to balance analytical depth with practical utility.(7) Thematic Network Analysis involves identifying basic themes, clustering them into broader organizing themes, and integrating these into overarching global themes that capture the core meaning of the data.(8) We adhered to the main analytical steps (1) coding the data; (2) identifying basic themes by organizing codes into coherent categories; (3) organizing themes by moving from coded excerpts to a more abstract level of meaning-making; (4) constructing global themes through iteratively returning to and refining the data; and (5) describing and interpreting relationships between themes.

In our analysis this meant that, for instance, the ‘container-code’ *Values & Attitudes* needed sub-coding by SB to explore whether the preliminary themes developed through group discussions could be supported. During this process, group-codes representing preliminary themes were added, and sub-codes were added and organized under these themes. At a certain point, efforts to fully sub-code all descriptive and structural codes with more substantial codes to explore further meaning behind excerpts proved unproductive. Therefore, we limited sub-coding and re-categorization. To gain a fresh perspective, all patient trajectories were displayed visually (Appendix F), preliminary themes were temporarily set aside, and brainstorming was conducted using the summaries of the participants’ stories to identify themes. SB examined how preliminary themes were still visible in the data, how themes related to each other, if new patterns came up and which themes were substantiated also by fieldnotes. This iterative and relational analysis supported the identification of new themes and refinement of preliminary discussed themes. This approach reflects a tailored, flexible method that allowed for both systematic and descriptive coding and creative, data-driven exploration of the material.

Throughout the analytic process, fieldnotes were used to complement and validate the findings, helping to inform, substantiate or question themes. By integrating insights from fieldnotes with the coded material, the research team was able to maintain a richer, context-sensitive understanding of each patient and family caregiver story. Additionally, fieldnotes highlighted ways in which fieldworkers may have influenced the data, capturing how they perceived participants and potentially shaped interactions, thereby contributing to reflexivity throughout data collection and the analysis.

A methodological challenge was that SB, while familiar with the transcripts across all country datasets (having reviewed coded transcripts across countries), was variably familiar with the material. Having conducted and coded the Dutch interviews herself, she was most familiar with this dataset. Not having had the experience of interviewing patients from other countries, and having to work with translated transcripts (from Portugal and Uganda) was more challenging than reading transcripts in original language (the US transcripts). Additionally, SB’s familiarity with the Dutch healthcare system meant that data from other countries sometimes appeared more striking or novel to her. This was an ongoing point of reflection within the team, helping her to discern whether observed differences were genuinely meaningful or primarily the result of SB’s frame of reference.

*5. Ethical considerations*

The study involved individuals in a vulnerable position across EU and non-EU countries. Local researchers executing the study were trained through interview role-play and supervised by senior research team members in their respective country. Personal and medical needs of patients and family caregivers took priority over the collection of data. Missed events and developments were reconstructed by the researcher by talking to the patient and family caregiver whenever the circumstances permitted this.

**References**

1. Namukwaya E, de Sousa AB, Lopes S, Touwen DP, van der Steen JT, Bélanger E, et al. EOLinPLACE: an international research project to reform the way dying places are classified and understood. Palliat Care Soc Pract. 2024;18:26323524231222498.

2. Braun V, Clarke V. Using thematic analysis in psychology. Qualitative research in psychology. 2006;3(2):77–101.

3. Pilarska J. 6 The Constructivist Paradigm and Phenomenological Qualitative Research Design. In: Anja P, Josephine P, Allison A, editors. Research Paradigm Considerations for Emerging Scholars. Bristol, Blue Ridge Summit: Channel View Publications; 2021. p. 64–83.

4. (WHO) WHO. International Classification of Diseases, Eleventh Revision (ICD-11) 2019/2021 [Available from: <https://icd.who.int/browse11>. Licensed under Creative Commons Attribution-NoDerivatives 3.0 IGO licence (CC BY-ND 3.0 IGO).

5. Saldaña J. The Coding Manual for Qualitative Researchers: SAGE; 2021.

6. Pelto PJ. Applied ethnography: Guidelines for field research: Routledge; 2016.

7. Guest G, MacQueen K, Namey E. Applied Thematic Analysis. Thousand Oaks, California: SAGE Publications, Inc.; 2012. Available from: <https://methods.sagepub.com/book/mono/applied-thematic-analysis/toc>.

8. Attride-Stirling J. Thematic networks: an analytic tool for qualitative research. Qualitative Research. 2001;1(3):385–405.
