# Supplementary material for: How Preferences and Reality on Where We Die Unfold: A Four‐Country Longitudinal Qualitative Study (EOLinPLACE)
Source: Health Expect. 2026 Jul 3;29(4):e70732. doi: 10.1111/hex.70732 (PMC13332329; doi:10.1111/hex.70732)
Supplement: Supplementary file 3 — Supporting File 3 [file HEX-29-e70732-s002.docx]

**Appendix C: Detailed information of researchers**

**Article title**: How Preferences and Reality on Where We Die Unfold: A Four-Country Longitudinal Qualitative Study (EOLinPLACE)

The team of fieldworkers consisted of:

- **MSc. B. Sanguedo**, BSc in Psychology (in Portugal), MSc in Psychology with a specialization in Clinical and Health Psychology (in Portugal). She undertakes her PhD as part of the EOLinPLACE Project. She is based in Portugal.
- **MSc. D. Olet**, MBChB (in Uganda) and Master in Public Health Methodology (in Belgium). She undertakes her PhD as part of the EOLinPLACE Project. She is also palliative care lecturer, supervising under- and postgraduate students in palliative care (in Uganda). She is based in Uganda.
- **MSc. I.D. da Silva**, BSc in Psychology (in Portugal) and MSc in Economic and Consumer Psychology (in the UK). She undertakes her PhD as part of the EOLinPLACE project and is also project manager. She is based in Portugal.
- **Ms. K.A. Eckels**, BSc in occupational sciences (in the US) and MOT in occupational therapy (OTR/L) (in the US). She is an occupational therapists at the Children’s Mercy Hospital and is an assistant researcher for the EOLinPLACE Project, undertaking her PhD at the University of Kansas Medical Center. She is based in the US.
- **Dr. M. Delilabera**, BSc in Psychology (in Brazil), MSc in Palliative Care (in Portugal), and a PhD in Health Psychology (in Portugal). She is a clinical and health psychologist and postdoctoral researcher for the EOLinPLACE Project. She is based in Portugal.
- **MSc. S.H. van de Beek**, BSc in Health Sciences (in the Netherlands) and MSc in Vitality and Ageing (in the Netherland). She undertakes her PhD at the Leiden University Medical Centre (LUMC) as part of the EOLinPLACE Project. She is based in the Netherlands.

The team was supervised by senior researchers from the EOLinPLACE Project and physicians from the palliative care teams who helped recruit participants for the study. They are experts in the respective national palliative care domain and the international scientific community.

- **D.P. Touwen PhD** is associate professor in Medical ethics at the Department of Medical Ethics and Health Law at the Leiden University Medical Center (in the Netherlands). In the EOLinPLACE Project, Dorothea is collaborator and local co-PI of the Netherlands (where she is based), and she leads the ethnographic work package of the project. She is supervisor of S.H. van de Beek.
- **Dr. B. Gomes** is coordinating researcher (equivalent to full professor) at the University of Coimbra and principal investigator of the EOLinPLACE Project. She is based in Portugal.
- **Dr. R. Garcia** is a palliative care physician and he leads the hospital palliative care team at Coimbra Health – Integrated Delivery System. He is based in Portugal.
- **Y.M. van der Linden MD PhD** is a radiation oncologist, professor of Palliative Medicine and she leads the Centre of Expertise in Palliative Care at the LUMC (in the Netherlands, where she is based). She is supervisor of S.H. van de Beek.
- **Dr. J.T. van der Steen** is associate professor at the Department of Public Health and Primary Care at the LUMC and senior researcher at Radboud university medical center (in the Netherlands). In the EOLinPLACE Project, she is collaborator and local co-PI of the Netherlands, where she is based.
- **Dr. E. Namukwaya** is a palliative care physician of the palliative care team at Mulago National Referral Hospital, combined with research and teaching activity. In EOLinPLACE she is collaborator and local co-PI in Uganda and leads the content validity-study of the proposed international classification of dying places. She is based in Uganda.
- **Dr. J. Brooks** is associate professor at the Department of Population Health at KUMC (in the US). In EOLinPLACE, she is collaborator and local co-PI in the US, where she is based.

Supervising researchers and physicians did not have an existing relationship with participants prior to the study except for Dr. R. Garcia who personally identified participants for the study, and Y.M. van der Linden MD PhD, who was familiar with and referred to one patient before the study started.

More information about the authors can be found at www.eolinplace.com.
