# Supplementary material for: How Preferences and Reality on Where We Die Unfold: A Four‐Country Longitudinal Qualitative Study (EOLinPLACE)
Source: Health Expect. 2026 Jul 3;29(4):e70732. doi: 10.1111/hex.70732 (PMC13332329; doi:10.1111/hex.70732)
Supplement: Supplementary file 4 — Supporting File 4 [file HEX-29-e70732-s006.docx]

**Appendix D: Interview topic guides**

**Article title**: How Preferences and Reality on Where We Die Unfold: A Four-Country Longitudinal Qualitative Study (EOLinPLACE)

**1. PATIENTS AGED 16 YEARS AND OVER**

**Interview details**

Interviewer, date of interview, location, time

**Participant details**

Participant’s ID number, basic socio-demographic and clinical characteristics

1. **PLACE OF CARE: PATIENT ISSUES**
2. **Narrative of trajectory of participant’s places of care since diagnosis, with attention to:**

- Transitions
- Places of care, with attention to nuances in places (e.g. places within places such hospital wards, own home vs. home of a relative/friend)

1. **Narrative of current place(s) of care, with attention to:**

- Description, characterisation and the meaning of place(s)
- Experiences

1. **Preferences of place of (actual) care, with attention to:**

- Place(s) of preference: In ideal circumstances
- Place(s) of preference: In actual circumstances

1. **PLACE OF CARE: CARER ISSUES**
2. **Narrative related to informal carer, with attention to:**

- Care role

1. **Agreement on place of care, with attention to:**

- Agreement with patient on preferences of place of care
- Agreement with patient on experiences of place of care

1. **Informal carers’ network**

- Support from others
- Decision-making

1. **FUTURE CARE**
2. **Narrative of places of future care for the patient, with attention to:**

***Care***

- Patients’ expectations and scenarios regarding the places they may need to visit or stay in when their condition and health worsens
- Preference of places depending on different scenarios

***Dying***

*First, the readiness and acceptability of addressing this topic with the patient and family will be discussed with the health care professionals responsible for the patient. The explicit mentioning of death and dying may not be acceptable to the patient and/or family. Several other ways of communication about dying will be explored, and verified with others that someone is ready to breach this issue.*

*The following question was suggested by health professionals in palliative care:*

“Where do you wish to be if your condition and health gets worse?”

*If acceptable to discuss, the following topics will be of relevance:*

1. **Preferences of place of death, with attention to:**

- Place(s) of preference: In ideal circumstances
- Place(s) of preference: In actual circumstances

1. **Patient-carer agreement on place of death, with attention to:**

- Agreement on preferences of place of death
- Agreement on suitability of place of death

1. **Decision-making**

- Participants in decision-making
- Concurrence and divergence on preferences
- Barriers and facilitators of choice

**11. Values**

- What constitutes a good life for this person? “What’s most important to him/her?” What makes life worth living?
- What constitutes good quality of dying for this person?
- How do these values figure in the care that s/he received, receives and will receive in the future?
- With whom does s/he talk about these issues?

**2. PATIENTS AGED 6 TO 15 YEARS**

*The interviews with patients aged 6 to 15 years will be conducted in the same flexible way as the patients aged 16 years and over, but adapted to the participant age and development stage. The advice and wishes by the participants on how and where the interview will be conducted will be followed. Their parents/legal guardians and professional carers will advise on the acceptability of the questions beforehand.*

*The same questions as those formulated in interview topic guide for patients aged 16 years and over will be of relevance for this topic guide. However, we will reduce it to a few basic questions that address the main research questions, to minimise the interview burden and adjust to the development stage of the child or adolescent. They will only approach place of care, not place of death. The remaining issues of relevance relating to carers, agreement with others and decision-making will be addressed in the interview with parents/legal guardians.*

**Interview details**

Interviewer, date of interview, location, time

**Participant details**

Participant’s ID number, basic socio-demographic and clinical characteristics

1. **PLACE OF CARE**
2. **Narrative of trajectory of participant’s places of care since diagnosis, with attention to:**

- Transitions
- Places of care, with attention to nuances in places (e.g. places within places such as where in hospital, own home or the home of a relative/friend)

1. **Narrative of current place(s) of care, with attention to:**

- Description, characterisation and meaning of place(s)
- Experiences

1. **Preferences of place of (actual) care, with attention to:**

- Place(s) of preference: In ideal circumstances
- Place(s) of preference: In actual circumstances

1. **Decision-making**

- Participants in decision-making
- Communication about preferences with parents/legal guardians, clinicians

**3. FAMILY CARERS**

**Interview details**

Interviewer, date of interview, location, time

**Participant details**

Participant’s ID number, basic socio-demographic characteristics

1. **CARING**
2. **Narrative of a typical day of caring for patient, with attention to:**

- Level of independence of patient
- Type of care provided
- Experiences of caregiving

1. **Support for carer**

- Carer’s informal network
- Professional carer(s)

1. **PLACE OF CARE**
2. **Narrative of trajectory of participant’s places of care since diagnosis, with attention to:**

- Transitions
- Places of care

1. **Narrative of current place(s) of care, with attention to:**

- Description, characterisation and meaning of place(s)
- Experiences

1. **Preferences of place of (actual) care, with attention to:**

- Place(s) of preference: In ideal circumstances
- Place(s) of preference: In actual circumstances

1. **Patient-carer agreement on place of care, with attention to:**

- Agreement on preferences of place of care
- Agreement on experiences of place of care

1. **Decision making, with attention to:**

- Participants in decision-making
- Concurrence and divergence on preferences

1. **FUTURE CARE**
2. **Narrative of places of future care for the patient, with attention to:**

***Care***

- Patients’ expectations and scenarios regarding the places they may need to visit or stay in when their condition and health worsens
- Preference of places depending on different scenarios

***Dying***

*First, the readiness and acceptability of addressing this topic with the patient and family will be discussed with the health care professionals responsible for the patient. The explicit mentioning of death and dying may not be acceptable to the patient and/or family. Several other ways of communication about dying will be explored, and verified with others that someone is ready to breach this issue.*

*The following question was suggested by health professionals in palliative care:*

“Where do you wish the patient to be if his/her health and condition gets worse?”

*If acceptable to discuss, the following topics will be of relevance:*

1. **Preferences of place of death, with attention to:**

- Place(s) of preference: In ideal circumstances
- Place(s) of preference: In actual circumstances

1. **Patient-carer agreement on place of death, with attention to:**

- Agreement on preference of place of death
- Agreement on suitability of place of death

1. **Decision making**

- Participants in decision-making
- Concurrence and divergence on preferences

1. **Values**

- What constitutes a good life for the patient? “What’s most important to him/her?” What makes life worth living? And for the carer?
- What constitutes good quality of dying for this person?
- How do these values figure in the care process?
- Do you talk about these issues?
