# Supplementary material for: How Preferences and Reality on Where We Die Unfold: A Four‐Country Longitudinal Qualitative Study (EOLinPLACE)
Source: Health Expect. 2026 Jul 3;29(4):e70732. doi: 10.1111/hex.70732 (PMC13332329; doi:10.1111/hex.70732)
Supplement: Supplementary file 5 — Supporting File 5 [file HEX-29-e70732-s005.docx]

**Appendix E: Codebook**

**Article title**: How Preferences and Reality on Where We Die Unfold: A Four-Country Longitudinal Qualitative Study (EOLinPLACE)

**Table 1.** Overview of the codes and group codes.

| **No.** | **Codes** | **Group code** |
| --- | --- | --- |
| **1.** | Actual place of care | Dying places |
| **2.** | Actual place of death |  |
| **3.** | Preferred place of care |  |
| **4.** | Preferred place of death |  |
| **5.** | (In-)congruence between preferred and actual dying places |  |
| **6.** | Care transition |  |
| **7.** | Emergency departments |  |
| **8.** | Sorting through options (e.g., ideal vs. realistic preferences) |  |
| **9.** | Dying places_other |  |
| **10.** | (In-)congruence in views on / preferences for places | Dynamics between patient & third party |
| **11.** | Informal caregiver (not) included in the decision-making process |  |
| **12.** | Discussing actual and preferred dying places |  |
| **13.** | Dynamics between patient & third party_other |  |
| **14.** | Practical logistics | Factors of influence on dying places |
| **15.** | Physical (In-)dependency |  |
| **16.** | Care capacity |  |
| **17.** | Access to EoL care services |  |
| **18.** | (Avail)ability of informal caregivers |  |
| **19.** | (Predicted) trauma of death at a certain place for bereaved family/loved ones |  |
| **20.** | Quality of care |  |
| **21.** | Treatments & symptom management |  |
| **22.** | Healthcare system structure |  |
| **23.** | Being (un)informed |  |
| **24.** | Values & Attitudes |  |
| **25.** | Diagnosis |  |
| **26.** | Factors of influence on dying places_other |  |
| **27.** | Feelings & Emotions | |
| **28.** | Coping strategies | |
| **29.** | Rural/Urban | |
| **30.** | Instances of decision-making | |
| **31.** | Agency | |
| **32.** | Other | |

**Table 2.** Sub-codes from the code group ‘dying places’ defined

| 1. | Code name | Actual place of care |
| --- | --- | --- |
|  | *Source of code* | Inductive (Research aim & objectives) |
|  | *Definition* | The physical location where the patient receives/received care |
|  | *When to use* | The code refers to any excerpts that refer to the actual location where someone received care. Use when the care setting is explicitly stated. Also use when someone explicitly states the length of stay |
|  | *When NOT to use* | Do not use this code when preferences or plans for future locations are being discussed (use ‘Preferred place of care’-code) |
|  | *Example* | Informal caregiver*: ‘We always took care of her at home, but now she went to a hospice’* |
|  | *Example when not to use* | Informal caregiver*: ‘We want her to go to the hospice’* |
|  | | |
| 2. | Code name | Actual place of death |
|  | *Source of code* | Inductive (Research aim & objectives) |
|  | *Definition* | The physical location where the patient died |
|  | *When to use* | The code refers to any excerpts that refer to the actual location where someone died. Use when the care setting is explicitly stated. |
|  | *When NOT to use* | Do not use this code when preferences or plans for future locations are being discussed (use ‘Preferred place of death’-code) |
|  | *Example* | Informal caregiver*: ‘Even though she wanted to die at home, she eventually died in a hospice’* – use ‘actual place of death’ & ‘preferred place of death’ & ‘(in-)congruence between preferred and actual dying places’ |
|  | *Example when not to use* | Informal caregiver*: ‘We want her to go to the hospice’* – use ‘preferred place of death’ |
|  | | |
| 3. | Code name | Preferred place of care |
|  | *Source of code* | Inductive (Research aim & objectives) |
|  | *Definition* | The preferred location to receive care |
|  | *When to use* | When the preferred PoC is mentioned in excerpts, either from the patient’s perspective or from the informal caregiver perspective. Do also include discussions about ‘not having a preference’ for place of care. Reasons why people prefer a place.. |
|  | *When NOT to use* | Don’t use for non-location related preferences (e.g. symptom management). If the transcript shows considerations between places rather than preferences, maybe think of using ‘sorting through options’ or both if in doubt. |
|  | *Example* | Patient*: ‘In the ideal scenario, I want to be cared for at home’* |
|  | *Example when not to use* | Patient: ‘*I am thinking of going to a hospice’ -* Use ‘sorting through options’ / Informal caregiver: *‘She is at the hospital now’* |
|  | | |
| 4. | Code name | Preferred place of death |
|  | *Source of code* | Inductive (Research aim & objectives) |
|  | *Definition* | The preferred location to die |
|  | *When to use* | When the preferred location for death is mentioned in excerpts, either from the patient’s perspective or from the informal caregiver perspective. Do also include discussions about ‘not having a preference’ for place of death. Reasons why people prefer a place. |
|  | *When NOT to use* | Don’t use for non-location related preferences (e.g. which coffin). If the transcript shows considerations between places rather than preferences, maybe think of using ‘sorting through options’ or both if in doubt. |
|  | *Example* | Patient*: ‘In the ideal scenario, I want to die at home’* |
|  | *Example when not to use* | Patient: *‘I want to be cared for at the hospital because I am afraid to feel like I am short of breath’* |
| 5. | Code name | (In-)congruence between preferred and actual dying places |
|  | *Source of code* | Literature(1) & Inductive (Research aim & Objectives & Data Familiarization) |
|  | *Definition* | Preferred and actual place align or don’t align |
|  | *When to use* | All excerpts that refer to either the alignment between actual & preferred place or non-alignment. So both. Don’t worry if there is a (non-)alignment across the serial interviews of one patient. Dorothy and Sifra will make sure to capture this during analysis. |
|  | *When NOT to use* | When someone only refers to preferred or actual place of care without explicitly mentioning if their situation aligns with what they wanted. |
|  | *Example* | Patient discusses a care transition that happened: ‘*In the end I am happy that I was transferred to the hospital to control my symptoms, even though I initially said not to want to go to the hospital*.’ |
|  | *Example when not to use* | Informal caregiver: ‘*I am happy that she [patient] is home’* |
|  | | |
| 6. | Code name | Care transition |
|  | *Source of code* | Inductive (Research aim & Objectives & Data Familiarization) |
|  | *Definition* | Transfer of a patient from one place of care to another |
|  | *When to use* | Whenever someone clearly states they are going from one place to another. If possible, include the length of stay or the reason for transition. |
|  | *When NOT to use* | If someone wishes to go from one place to another, instead of it actually happening. If it is only for a day. |
|  | *Example* | Patient*: “I had to go and be admitted to the hospital to readjust my breathing support since I had trouble breathing.”* |
|  | *Example when not to use* | Patient*: “I had to go to the hospital for my checkup, it took an hour.”* |
|  | | |
| 7. | Code name | Emergency Departments |
|  | *Source of code* | Beatriz Sanguedo |
|  | *Definition* | Captures instances when the participant refers to the ED as a PoC/PoD, the journey there (i.e., ambulances as extended part from an ED), interventions and experiences within that place. Note: Depending on the country, other terms may be used (ED, emergency room, emergency care, A&E, etc.). |
|  | *When to use* | Every mention related to EDs as a PoC/PoD. Only code when explicitly talked about this place. It may capture care that took place at ED, decision making about visiting ED and/or preferences about this setting. |
|  | *When NOT to use* | When just referring to hospital admission or when the text refers to acute situations that are not related to the emergency department specifically. |
|  | *Example* | Patient*: “It was so bad, I had to go to the Emergency Department”* |
|  | *Example when not to use* | Patient: ‘I went to the hospital’ |
|  | | |
| 8. | Code name | Sorting through options |
|  | *Source of code* | Literature(2) & Inductive (Data Familiarization) |
|  | *Definition* | Discussing and considering options, related to dying places. The preference for dying places, based on the ideal way a disease trajectory could go. |
|  | *When to use* | Use to show when a patient / informal caregiver, explicitly talks about options for places of care/death, this includes considerations between the ‘ideal’ and the ‘realistic’ preferred/actual place. |
|  | *When NOT to use* | Don’t use this code always when someone discusses their preferences for places (then you might just need to use e.g., ‘preferred place of care). The person needs to talk about scenario’s or options that they would consider. |
|  | *Example* | Patient: *‘I would prefer to die at home, but I am also considering to go to a hospice if [name partner] cannot physically handle taking care of me anymore.’* |
|  | *Example when not to use* | Patient: *‘I am happy that I am home!’ (use ‘preferred place of care’)* |
|  | | |
| 9. | Code name | Dying places_other |
|  | *Source of code* | Usual code for group-codes |
|  | *When to use* | Use whenever you feel something should be coded within this code-group but there is no other appropriate sub-code. This code informs discussions on the codebook. |
|  | *When NOT to use* | When there is already a designated code. Once codebook is definitive, this code should not be used anymore. |

**Table 3.** Sub-codes from the code group ‘dynamics between patients & third parties’ defined

| 10. | Code name | (In-)congruence in views on / preferences for places |
| --- | --- | --- |
|  | *Source of code* | Inductive (Research Aim & Objectives) |
|  | *Definition* | Whenever it is clear that there is a (non-)alignment in views on certain places or if preferences align/differ between patient and informal caregiver. |
|  | *When to use* | Whenever there is talk of, or between patient and family members’ communication about preferred and/or actual dying places. Might be used in a joint interview, when there is a discussion that shows some friction/conflict. |
|  | *When NOT to use* | Don’t use when not about dying places and when a patients’ views/preference on/for places start to change. It is about patient & informal caregiver dynamics. |
|  | *Example* | Patient: ‘*You constantly want to have him [son] home, but I feel so much safer in a hospital*.’ Informal caregiver: ‘*Well don’t blame me for thinking the hospital is such a cold and stressful place*…’ |
|  | *Example when not to use* | Patient: ‘*I feel like [name] wants me to go for treatment to extend life*.’ |
|  | | |
| 11. | Code name | Informal caregiver (not) included in the decision-making process |
|  | *Source of code* | Inductive (data familiarization) |
|  | *Definition* | Informal caregiver (often family member) does either feel included or not included during instances of decision making. |
|  | *When to use* | Use this code when it is discussed if informal caregivers feel included in decision-making processes and conversations about dying places. |
|  | *When NOT to use* | Don’t use this code when the informal caregiver generally feels/does not feel included in the decision making process about things other than places. |
|  | *Example* | Informal caregiver: “*I really don’t feel like I can express my needs here. I, for instance, am truly afraid my back won’t be able to handle the care anymore. And then? But when we talk about this, the physician only focusses on [name patient].”* |
|  | *Example when not to use* | Patient: “*We* [informal caregiver] *always discuss what we want the funeral to look like and what fits us both best around saying goodbye to my loved ones.”* |
|  | | |
| 12. | Code name | Discussing actual and preferred dying places |
|  | *Source of code* | Literature (1, 3) |
|  | *Definition* | Conversations about dying places and preferences. |
|  | *When to use* | Whenever interviewees talk about the discussions they have with others about (preferred/actual) dying places. An example can also be the reference to a formal conversation with healthcare professionals (e.g., advance care planning) |
|  | *When NOT to use* | When it isn’t about issues of the EOL |
|  | *Example* | Informal caregiver: ‘*The doctor asked us if you* [patient] *wanted to be resuscitated or go to the hospital in case of emergencies. We discussed and decided that we don’t want to go to the hospital anymore’* |
|  | *Example when not to use* | Patient to informal caregiver: *‘I want to stay at home as long as possible, right?*’ Informal caregiver: ‘*Yes, indeed’* (to be coded with ‘preferred PoC’) |
|  | | |
| 13. | Code name | Dynamics between patients and third parties_other |
|  | *Source of code* | Usual code for group-codes |
|  | *Definition* | - |
|  | *When to use* | Use whenever you feel something should be coded within this code-group but there is no other appropriate code. This code informs discussions on the codebook. |
|  | *When NOT to use* | When there is already a designated code. Once codebook is definitive, this code should not be used anymore. |

**Table 4.** Sub-codes from the code group ‘factors of influence on dying places’ defined

| 14. | Code name | Practical logistics | |
| --- | --- | --- | --- |
|  | *Source of code* | Literature (4) & Inductive (Data Familiarization) | |
|  | *Definition* | Anything on practical or organizational logistics such as transport (issues) | |
|  | *When to use* | Use this code when practical issues (e.g., transport) are discussed. | |
|  | *When NOT to use* | When it is not about preference to place | |
|  | *Example* | Patient: ‘*I want to stay at home, but need to sleep downstairs, but my living room is too small to put a hospital bed in*.’ | |
|  | *Example when not to use* | Patient: ‘*I didn’t really want to be in hospital’* | |
|  | | | |
| 15. | Code name | Physical (in-)dependency | |
|  | *Source of code* | Literature (5, 6) & Inductive (Data Familiarization) | |
|  | *Definition* | Patient being physically independent or dependent on others for care | |
|  | *When to use* | Use code when excerpt mentions that the patients’ physical (in-)dependency is part a reason to go to / prefer a place. | |
|  | *When NOT to use* | When not explicitly mentioned as part of an impact on actual or preferred dying place | |
|  | *Example* | Patient: ‘ *Since he [son] is getting worse and worse [referring to physical state], we do need to consider hospice admission to manage symptoms*.’ | |
|  | *Example when not to use* | Patient: ‘*I can get out of bed without support*.’ (does not state anything about the influence of this fact on the preferred or actual dying places). | |
|  | | | |
| 16. | Code name | Care capacity | |
|  | *Source of code* | Literature (5) & Inductive (Data Familiarization) | |
|  | *Definition* | *Availability of resources (human and material resources)* | |
|  | *When to use* | When resources needed and they are or are not available at the place of care. Also, when people need/want to go to a place but it’s not possible due to a lack of care capacity. When people are in a certain place and need certain care, but it’s not possible and therefore have to deal without this care. | |
|  | *When NOT to use* | Don’t use when it’s about care capacity in general, without focusing on the patient’s case (e.g., when patient discusses stories about early days when there was e.g., a lack of certain types of care). | |
|  | *Example* | Informal caregiver: ‘*We need home care, but we’ve been waiting for weeks. We do it ourselves now, but we are waiting for the home-care organization to tell us they can schedule us in.’* | |
|  | *Example when not to use* | Patient: ‘*The hospital I was taken to wasn’t appropriate’* | |
|  | | | |
| 17. | Code name | Access to EOL care services | |
|  | *Source of code* | Literature (5, 7) & Inductive (Data Familiarization) | |
|  | *Definition* | *Having or not having access to certain dying places* | |
|  | *When to use* | When (not) having access to EOL care is mentioned (e.g., financial struggles; to access a place). This always needs to be explicitly linked to dying places. | |
|  | *When NOT to use* | When it is not linked to or having impact on dying places | |
|  | *Example* | Patient: ‘*I need care which is only available in a hospital far away’* | |
|  | *Example when not to use* | Informal caregiver: ‘*I wish he would go to a hospice, but he wants to stay home*.’ | |
|  | | | |
| 18. | Code name | (Avail)ability of informal caregivers | |
|  | *Source of code* | Literature (1) & Inductive (Data Familiarization) | |
|  | *Definition* | The availability and ability (i.e., capability) of informal caregivers to provide support (e.g., financial, physical care, emotional support). | |
|  | *When to use* | When informal caregivers are (not) readily available when needed to provide certain types of support, or when about their capabilities to (not) do so. | |
|  | *When NOT to use* | When it’s not explicitly stated or when it is not about dying places. | |
|  | *Example* | Patient: ‘*My family is usually present whenever I need care at home’* | |
|  | *Example when not to use* | Patient: ‘*I required care which need me to be hospitalized.’* | |
|  | | | |
| 19. | Code name | (Predicted) trauma of death at a certain place for bereaved family/loved ones | |
|  | *Source of code* | Literature (4) | |
|  | *Definition* | (Anticipated) disturbances caused by death of a loved one in the places of care | |
|  | *When to use* | This code can be used when the excerpt makes mention of anticipated death during end-of-life care, and the way it might impact the bereaved family | |
|  | *When NOT to use* | When it is not about dying places and the (predicted) impact on the family | |
|  | *Example* | Patient: ‘*I would like to die from home but I am afraid of how my family will handle my demise and the way the feel in the house after it.’* | |
|  | *Example when not to use* | Patient: ‘*Due to crisis the informal caregivers took me to hospital.’* | |
| 20. | Code name | Quality of care | |
|  | *Source of code* | Literature (1) & Inductive (Data Familiarization) | |
|  | *Definition* | *The quality of care delivered in the formal healthcare system. This includes topics like the level of c*omfort (3, 4), symptom management (4), professional expertise (8), sustainability and e.g., aspects of continuity of care | |
|  | *When to use* | Use when the excerpt is referring to the type of care the patient received at a place, linked to dying places and their ideas about quality of care in this place. | |
|  | *When NOT to use* | Don’t use this code when it is about care provided by informal caregiver. | |
|  | *Example* | Patient: ‘*I like it at home, when the hospice team visits to control my pain’* | |
|  | *Example when not to use* | Patient: ‘*I hate it when my wife needs to help me shower*.’ | |
|  | | | |
| 21. | Code name | Treatments & Symptom management | |
|  | *Source of code* | Inductive (Data Familiarization) | |
|  | *Definition* | Medical care given a patient for an illness including presenting symptoms | |
|  | *When to use* | Whenever the excerpt mentions any form of medical care given to the patient to manage their symptoms but only when the treatment and/or symptom management has an impact on the place where someone can receive care/die. | |
|  | *When NOT to use* | Don’t code all excerpts about treatments. It should be related to the code-group (treatment as a factor that influences preferred & actual dying places). | |
|  | *Example* | Patient: ‘*I had to see the oncologist about stopping my chemotherapy then continue with total palliative care at home’* | |
|  | *Example when not to use* | Informal caregiver: ‘*I will get chemotherapy and after that, if it does not work anymore, there is one treatment left.’* | |
|  | | | |
| 22. | Code name | Healthcare system structure | |
|  | *Source of code* | Literature (5) & Inductive (Data Familiarization) | |
|  | *Definition* | Organizations, people and actions whose primary intent is to promote, restore or maintain health | |
|  | *When to use* | This code can be used when the excerpt mentions the system of care for patients (e.g., when specific to that country’s system) if it influences where people can go / prefer to go to receive care / die; outside of healthcare quality. | |
|  | *When NOT to use* | When it is not about preference to place | |
|  | *Example* | Informal caregiver: ‘*We do not want her to die at home, because if she dies at home, the police will first need to research if it was a natural death. Also, dealing with the deceased body is difficult. Hence, we prefer her to die in the hospital.* | |
|  | *Example when not to use* | Informal caregiver: ‘*The complications she developed can be managed by the home care team’ (too general, and not specific to certain aspects of your country’s healthcare system, influencing preferred/actual dying places*). | |
|  | | | |
| 23. | Code name | Being (un)Informed | |
|  | *Source of code* | Literature (6) | |
|  | *Definition* | Making choices based on good information and good understanding of this information (and thus good communication). | |
|  | *When to use* | Everything on achieving/making an informed choice. E.g., information provision / communication(4), ability to understand information (health literacy). Only use when specific to dying places. | |
|  | *When NOT to use* | If the informed choice is focused on treatment decisions and this is not (in)directly related to places at the EOL. | |
|  | *Example* | *‘I did not feel like I truly had the time to comprehend what the doctor told me and needed to make a decision anyways.’* | |
|  | *Example when not to use* | *‘I eventually chose to go home because I just thought it would be best for me’* (can be coded with ‘preferred place of care’ and ‘(non-)alignment’-code | |
|  | | | |
| 24. | Code name | Values & Attitudes | |
|  | *Source of code* | Literature (1, 3, 5) & Inductive (Data Familiarization) | |
|  | *Definition* | Anything that seems / is important to an informal caregiver and/or patient. This can be viewed as a value, but sometimes this is not explicitly stated as a value. Hence, you can including anything that is ‘important’ to this person (e.g., social support, social networks, (in-)formal emotional / practical support). | |
|  | *When to use* | To label discussion around what the patient and/or caregiver value or is important to them, only when about the EOL in relation to dying places. | |
|  | *When NOT to use* | When it’s not (in-)directly related to dying places. | |
|  | *Example* | Patient: ‘*I have received all the care and support needed during my illness and I am comfortable being taken care for at home’* | |
|  | *Example when not to use* | Patient: ‘*I love to go on holidays, but I don’t have the money’.* | |
| 25. | Code name | Diagnosis | |
|  | *Source of code* | Data familiarization | |
|  | *Definition* | The illness and illness progression of a patient. | |
|  | *When to use* | You can use this code if the diagnosis is a factor in play when discussing / choosing / preferring a certain dying place. | |
|  | *When NOT to use* | Don’t use when it’s just about the diagnosis, stories about illness burden, or stories about trajectory without explicit reference to dying places. | |
|  | *Example* | Patient: “*The physician also did not believe the illness would progress so quickly. They expect me to live for a few more weeks. So, I decided to go to a hospice.”* | |
|  | *Example when not to use* | Patient: “*I never thought to that it would be so restricting. I just feel like the pain cannot be managed with medicine anymore. It really is exhausting.”* | |
|  | | | |
| 23. | Code name | Factors of influence on dying places_other |  |
|  | *Source of code* | Usual code for group-codes |  |
|  | *Definition* | - |  |
|  | *When to use* | Use whenever you feel something should be coded within this code-group but there is no other appropriate code. This code informs discussions on the codebook. |  |
|  | *When NOT to use* | Once codebook is definitive, this code should not be used anymore. |  |

**Table 5.** Sub-codes not attached to a code group

| 27. | Code name | Feelings & Emotions |
| --- | --- | --- |
|  | *Source of code* | Literature (3-6) & Inductive (Data Familiarization) |
|  | *Definition* | Emotion: a state of awareness. Feeling: experiences as a result of emotions |
|  | *When to use* | When emotions and feelings are expressed in the quotation. |
|  | *When NOT to use* | Do not use when the emotions or feelings are not related to dying places |
|  | *Example* | Patient: *‘When I heard she could be home till the end, I felt much better’* |
|  | *Example when not to use* | Informal caregiver: ‘*Aggressive therapy was still needed.’* |
|  | | |
| 28. | Code name | Coping strategies |
|  | *Source of code* | Literature (3) & Inductive (Data Familiarization) |
|  | *Definition* | Coping strategy is an act, a series of actions, or thought process used in meeting a stressful or unpleasant situation |
|  | *When to use* | When ways of handling a stressful situation are mentioned; related to places |
|  | *When NOT to use* | When it is about physical strategies to ‘deal’ with symptoms. |
|  | *Example* | ‘Informal caregiver: *‘I always try to make jokes and lighten the mood when talking about these kinds of topics* [EOL]..*. Don’t know why...’* |
|  | *Example when not to use* | Informal caregiver: ‘*I tried to arrange for a volunteer to be with my wife, just so I can get some time to myself.’.* (no coping strategy but a practical solution). |
|  | | |
| 29. | Code name | Rural/Urban |
|  | *Source of code* | Team member (for her dissertation, this is her topic of interest) |
|  | *Definition* | Rural areas usually refer to villages and urban areas usually refer to cities, suburbs and towns |
|  | *When to use* | This code can be used when referring to the kind of setting (either rural or urban) where the patient is taken care of. |
|  | *When NOT to use* | When there is no explicit mention of place of care in terms of rural or urban |
|  | *Example* | Informal caregiver: ‘*We had to take* [patient] *from a big hospital in the city to his village home since he was more comfortable and wished to die there’* |
|  | *Example when not to use* | Informal caregiver: ‘[Name patient] *died in the hospital*.’ |
|  | | |
| 30. | Code name | Instances of decision-making |
|  | *Source of code* | Inductive (data familiarization) |
|  | *Definition* | Decision-making occurred between patients/informal caregivers/professionals |
|  | *When to use* | Whenever an interviewee mentions an instance where there were conversations around decision-making. Because we want to know who had conversations with who, if patients/informal caregivers have an influence on what happens, if they have a say and are included in conversations about dying places. |
|  | *When NOT to use* | When it is not about the EOL (specifically dying places) or when it’s more about having preferences rather than actual decision-making instances. |
|  | *Example* | Patient: ‘*Yesterday I heard that the bloodwork was okay, so then I decided to go for the hip replacement so I can move around in home again; assuming that the chemotherapy can be postponed for at least 2 months.’* |
|  | *Example when not to use* | Patient: ‘*I have been home for a week now’* |
|  | | |
| 31. | Code name | Agency |
|  | *Source of code* | Literature (5) & Inductive (data familiarization) |
|  | *Definition* | The belief that you (the patient/informal caregiver) have power / the ability to affect your future. Does the patient feel like they have options, they have influence, they have a choice? Or not having agency: not feeling like you can influence the choices, options, or e.g., an informal caregiver needs to make a choice. |
|  | *When to use* | When due to diagnosis, economic status, practical or personal issues patients and/or informal caregivers are unable to actively make a choice in their care trajectory. Do patients feel they have agency, they have a say, they can make a choice? Sometimes people feel there is no choice / agency even though there is and vice versa. This is interesting to capture. |
|  | *When NOT to use* | If the choice is not about dying places or things that (you feel) indirectly influences the issue of dying places. Also, don’t use if it’s just about the difficulty of making decisions or speaking about preferences. |
|  | *Example* | Informal caregiver: ‘*You know, he [father/patient] cannot go anywhere. He wants to stay home, but the only option is to go to the nursing home. A hospice as dying place is not an option because of his diagnosis [dementia*].’ |
|  | *Example when not to use* | Informal caregiver: ‘*He just wants to be home*.’ |
| 32. | Code name | Other |
|  | *Source of code* | Usually included to cover anything that is not covered with the codes so far. |
|  | *Definition* | Any other code related to place of care and place of death that hasn’t been mentioned among these codes |
|  | *When to use* | Use whenever you feel something cannot be coded with the codes existing in the codebook so far. Please make notes on what code you feel you would need. |
|  | *When NOT to use* | When there is already a designated code. Once codebook is definitive, this code should not be used anymore. |

**Reference list**

1. Nilsson J, Blomberg C, Holgersson G, Carlsson T, Bergqvist M, Bergström S. End-of-life care: Where do cancer patients want to die? A systematic review. Asia Pac J Clin Oncol. 2017;13(6):356–64.

2. Gomes B, Calanzani N, Gysels M, Hall S, Higginson IJ. Heterogeneity and changes in preferences for dying at home: a systematic review. BMC Palliat Care. 2013;12:7.

3. Driessen A, Borgstrom E, Cohn S. Placing death and dying: Making place at the end of life. Soc Sci Med. 2021;291:113974.

4. Noyes M, Herbert A, Moloney S, Irving H, Bradford N. Location of end-of-life care of children with cancer: A systematic review of parent experiences. Pediatr Blood Cancer. 2022;69(6):e29621.

5. Collier A, Broom A. Unsettling Place(s) at the end of life. Soc Sci Med. 2021;288:113536.

6. Sathiananthan MK, Crawford GB, Eliott J. Healthcare professionals' perspectives of patient and family preferences of patient place of death: a qualitative study. BMC Palliat Care. 2021;20(1):147.

7. Lin SC, Huang MC, Yasmara D, Wuu HL. Impact of palliative care on end-of-life care and place of death in children, adolescents, and young adults with life-limiting conditions: A systematic review. Palliat Support Care. 2021;19(4):488–500.

8. Papadatou D, Kalliani V, Karakosta E, Liakopoulou P, Bluebond-Langner M. Home or hospital as the place of end-of-life care and death: A grounded theory study of parents' decision-making. Palliat Med. 2021;35(1):219–30.
