# Supplementary material for: How Preferences and Reality on Where We Die Unfold: A Four‐Country Longitudinal Qualitative Study (EOLinPLACE)
Source: Health Expect. 2026 Jul 3;29(4):e70732. doi: 10.1111/hex.70732 (PMC13332329; doi:10.1111/hex.70732)
Supplement: Supplementary file 6 — Supporting File 6 [file HEX-29-e70732-s004.pdf]

These graphical representations of trajectories are visual syntheses of participant narratives- illustrating a patients' journey based on qualitative data rather than an objective representation or factual account.

Preferred place of care and death: home and/or hospice

The Netherlands

Family No. 1

	Patient	Family caregiver
Sex	Female	Male
Age	60-69 y/o	60-69 y/o
Illness	Cancer	N/A
Relationship	Partner	Partner

Outpatient visits

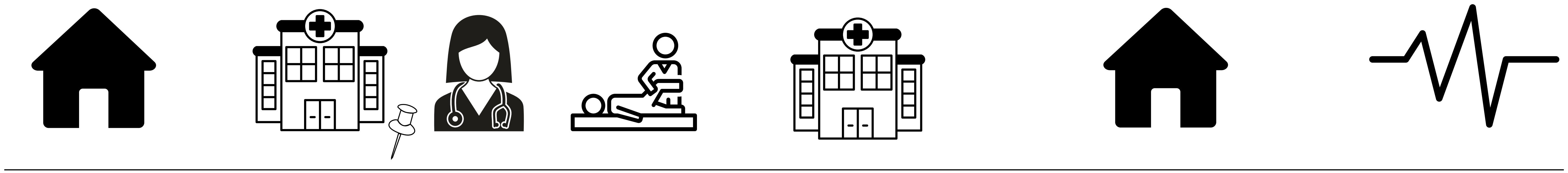

Home

Hospital admission: hip replacement

Home

Alive at end of study

Start of study

The Netherlands

Family No. 2

Preferred place of care and death: home

	Patient	Family caregiver
Sex	Female	Male
Age	50-59 y/o	60-69 y/o
Illness	Neuromuscular disease	-
Relationship	Married	

Outpatient visits

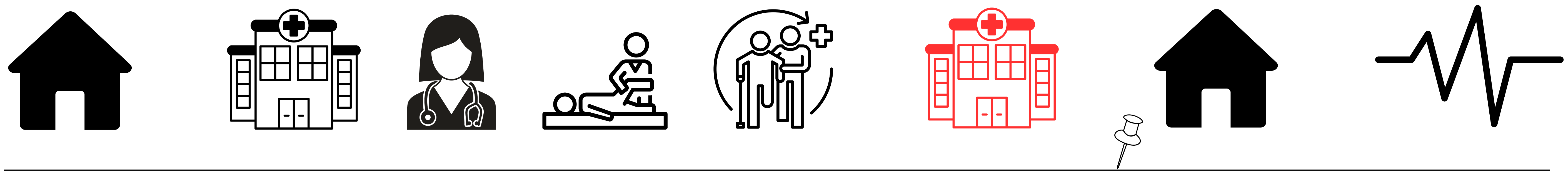

Home

Hospital exams, rehabilitation, and study treatments

ICU admission: readjusting breathing support

Home

Alive at end of study

Start of study

The Netherlands

Family No. 3

Preferred place of care and death: home

	Patient	Family caregiver
Sex	Male	Female
Age	70-79 y/o	70-79 y/o
Illness	Neuromuscular disease	N/A
Relationship	Married	

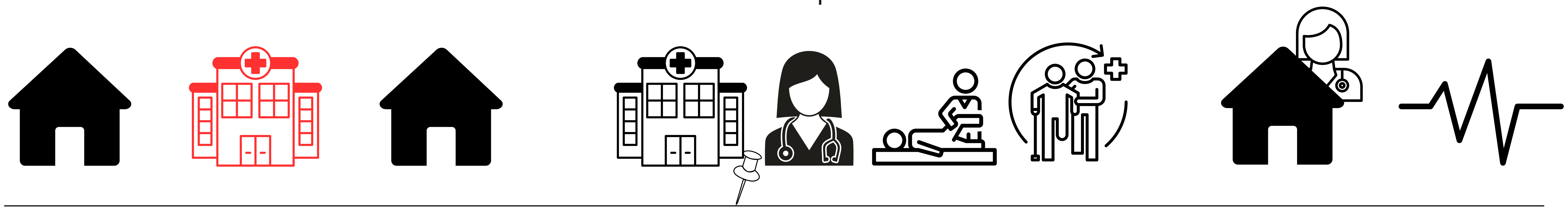

NETHERLANDS

Family No. 4

Preferred place of care and death: home & nursing home

	Patient	Family caregiver
Sex	Male	Female
Age	90-99 y/o	60-69 y/o
Illness	Dementia	N/A
Relationship	Father	Daughter

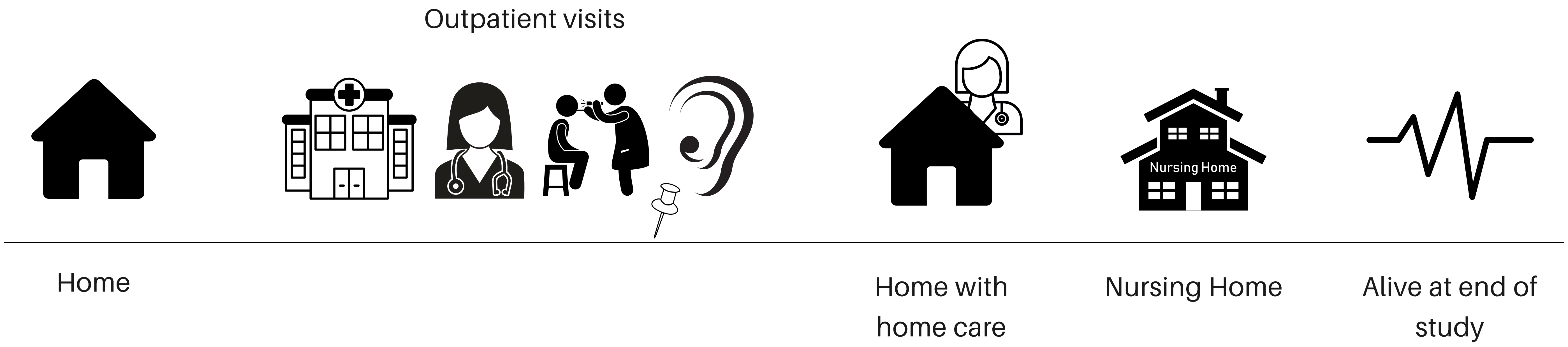

Start of study

PORTUGAL

Family No. 5

Preferred place of care and death: home

	Patient	Family caregiver
Sex	Female	Male
Age	50-59 y/o	50-59 y/o
Illness	Cancer	N/A
Relationship	Married	

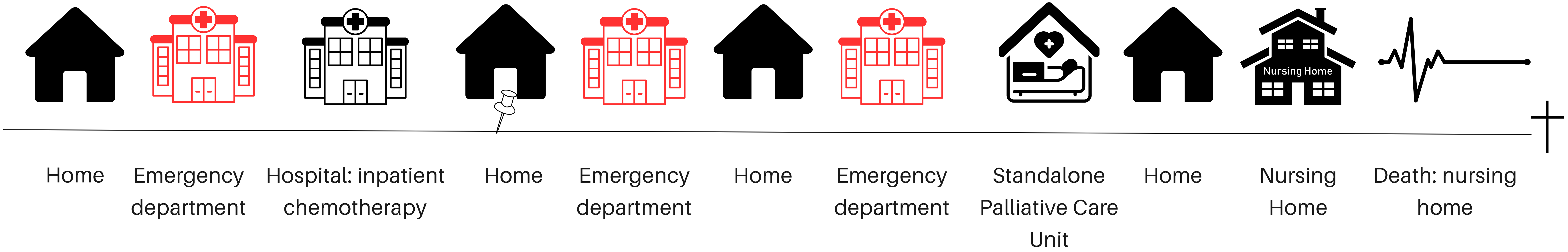

Start of study

PORTUGAL

Family No. 6

Preferred place of care: home
Preferred place of death: PC unit

	Patient	Family caregiver
Sex	Female	Male
Age	60-69 y/o	60-69 y/o
Illness	Cancer	N/A
Relationship	Married	

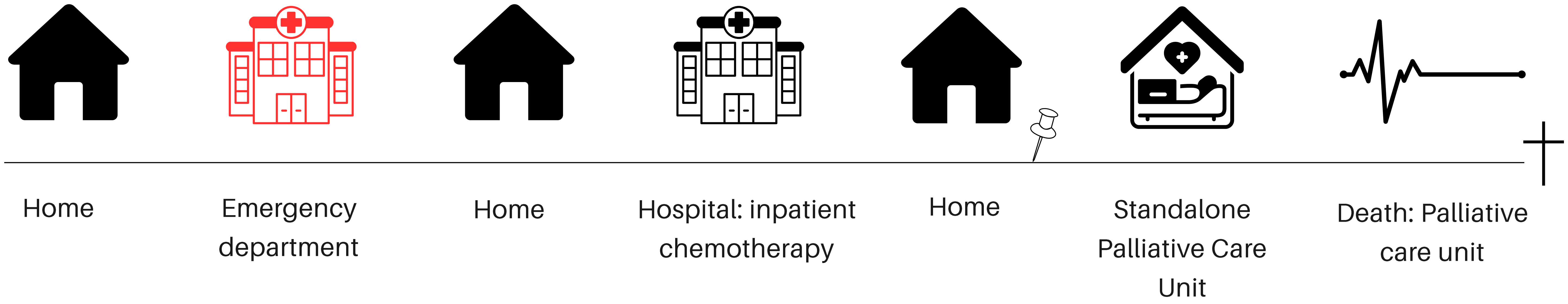

Start of study

PORTUGAL

Family No. 7

Preferred place of care: home
Preferred place of death: PC unit

	Patient	Family caregiver
Sex	Male	Female
Age	70-79 y/o	30-39
Illness	Heart- and cerebrovascular disease	N/A
Relationship	Father	Daughter

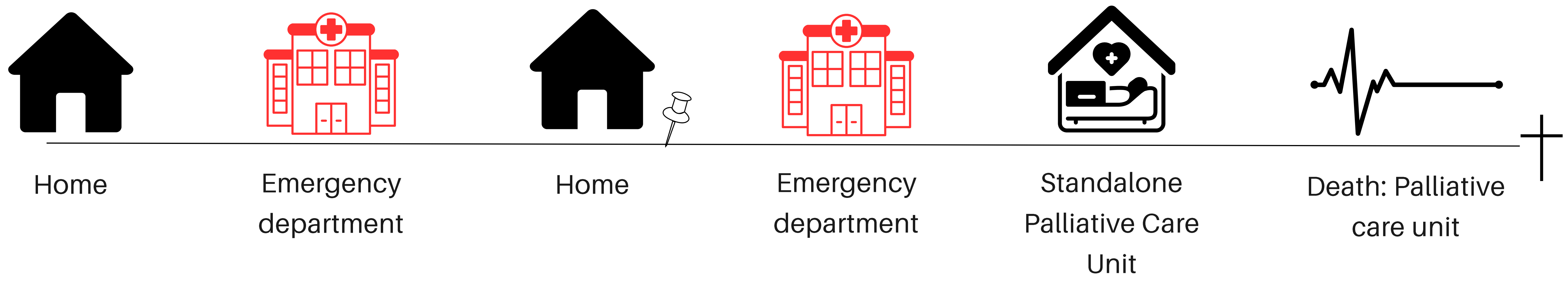

Start of study

UGANDA

Family No. 8

Preferred place of care and death: home

	Patient	Family caregiver
Sex	Female	Male
Age	80-89 y/o	20-29 y/o
Illness	Cardiac decompensation	N/A
Relationship	Grandmother	Grandson

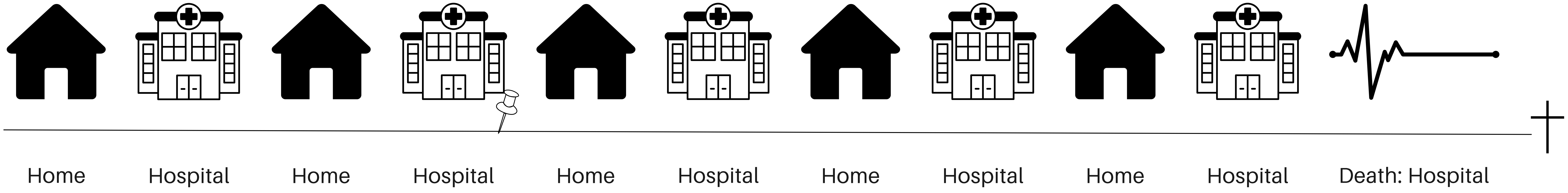

Start of study

UGANDA

Family No. 9

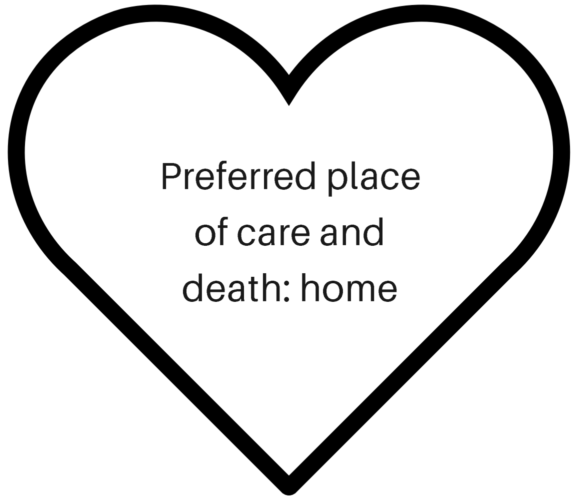

	Patient	Family caregiver
Sex	Male	Male
Age	60-69 y/o	30-39 y/o
Illness	Cancer	N/A
Relationship	Father	Son

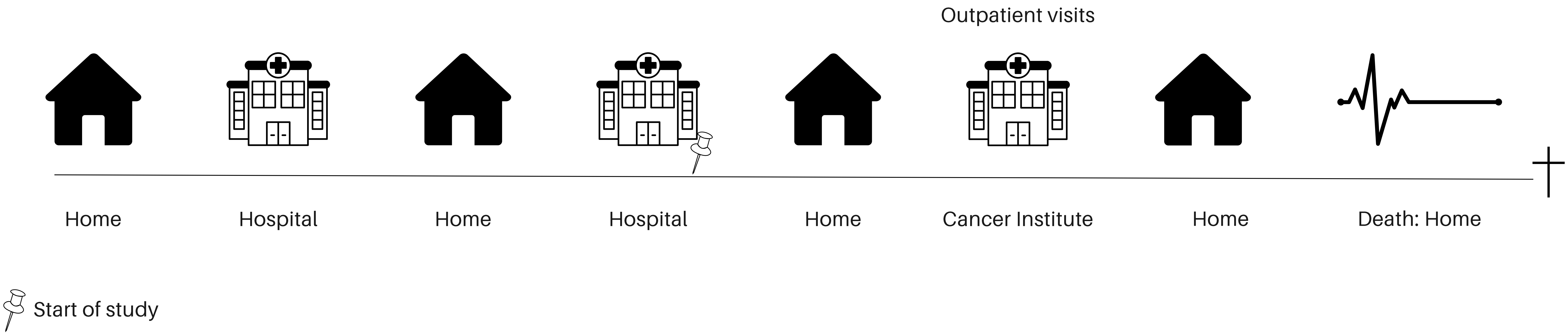

UGANDA

Family No. 10

Preferred place of care and death: unknown

	Patient	Family caregiver
Sex	Female	Female
Age	70-79 y/o	40-49 y/o
Illness	Dementia	N/A
Relationship	Aunt	Niece

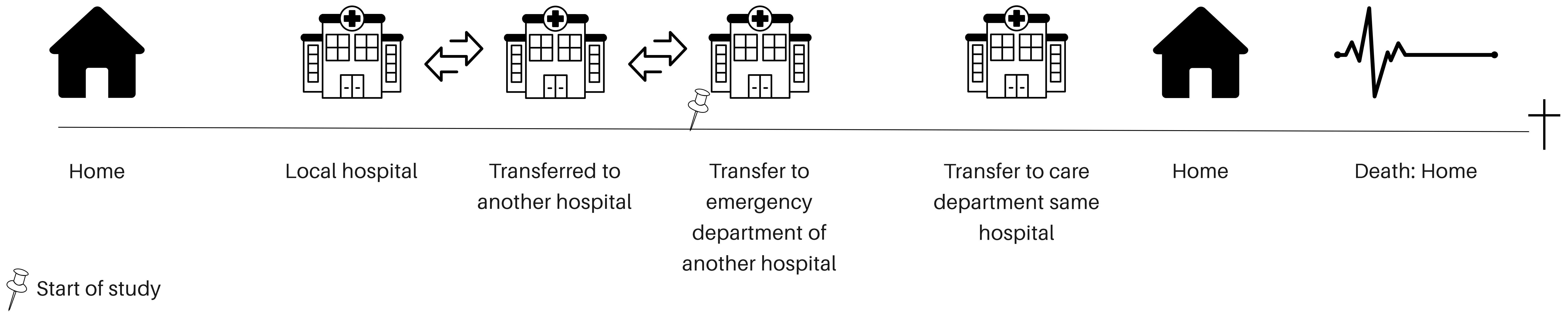

UGANDA

Family No. 11

Preferred place of care and death: home

	Patient	Family caregiver
Sex	Male	Male
Age	30-39 y/o	40-49 y/o
Illness	Heart- and cerebrovascular disease	N/A
Relationship	Friends	

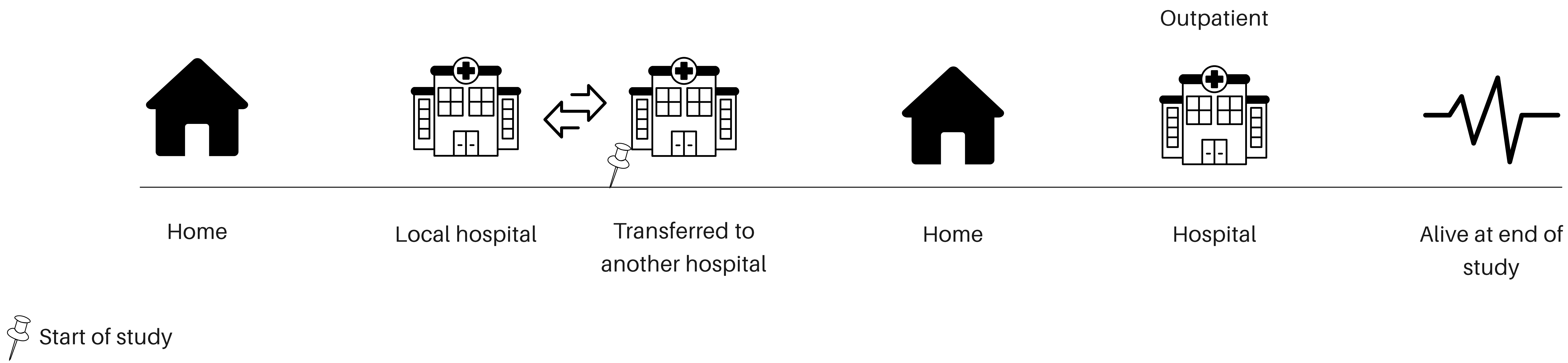

The United States

Family No. 12

Preferred place of care and death: home & hospice house

	Patient	Family caregiver
Sex	Female	Female
Age	70-79 y/o	Not reported
Illness	Cancer	N/A
Relationship	Aunt	Niece

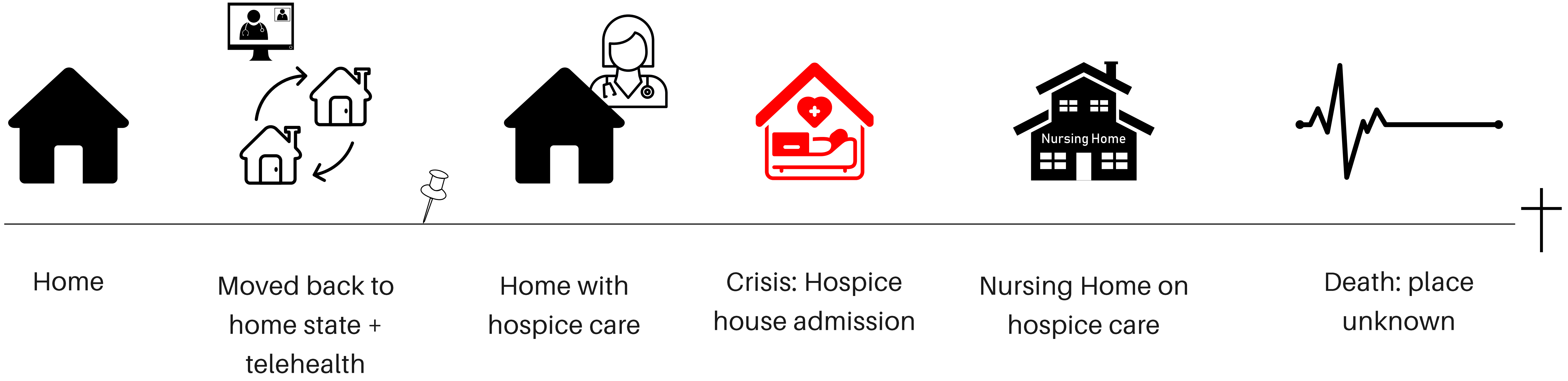

Start of study

The United States

Family No. 13

Preferred place of care and death: home

	Patient	Family caregiver
Sex	Male	Female
Age	40-49 y/o	Not reported
Illness	Neuromuscular disease	N/A
Relationship	Partner	Partner

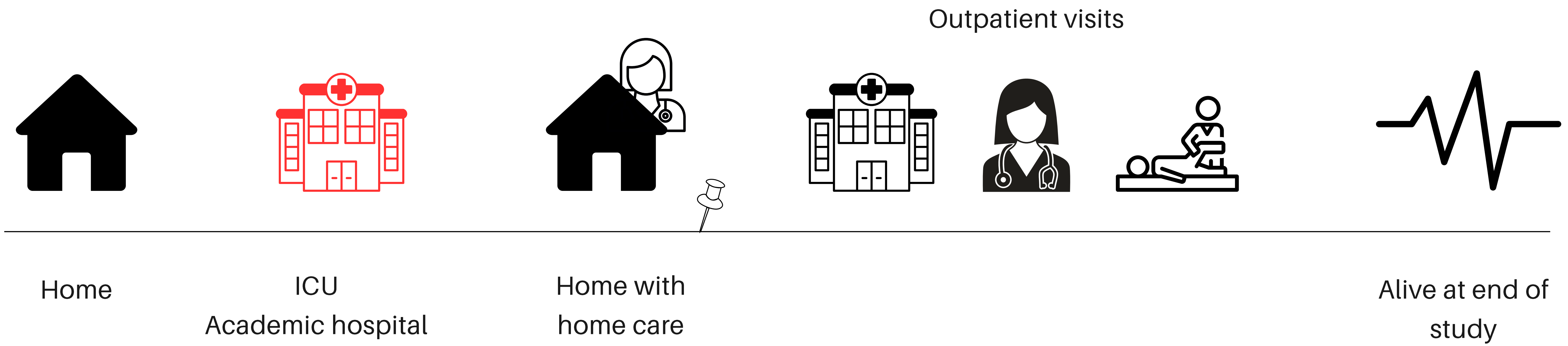

Start of study

The United States

Family No. 14

Preferred place of care: home & a further away hospital

	Patient	Family caregiver
Sex	Female	Male
Age	60-69 y/o	Not reported
Illness	Cancer	N/A
Relationship	Partner	Partner

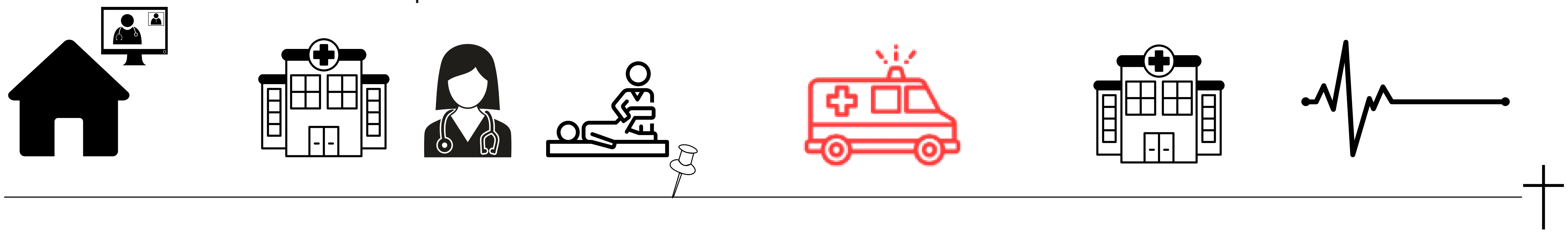

Start of study
